# Synthesis, In Vitro, and In Vivo Investigations of Pterostilbene-Tethered Analogues as Anti-Breast Cancer Candidates

**DOI:** 10.3390/ijms241411468

**Published:** 2023-07-14

**Authors:** Guoxun Li, Jian Li, Wenqian Wang, Xiaoqing Feng, Xingkang Yu, Shuo Yuan, Wei Zhang, Jialing Chen, Caijuan Hu

**Affiliations:** 1 School of Pharmacy, Changzhou University, Changzhou 213164, China; liguoxuner@126.com (G.L.); lijianchem@cczu.edu.cn (J.L.); 15695186052@163.com (W.W.); fxqfw@cczu.edu.cn (X.F.); s21080917007@smail.cczu.edu.cn (X.Y.); s21090860003@smail.cczu.edu.cn (S.Y.); weiiiiii02212023@163.com (W.Z.); 19851990167@163.com (J.C.); 2 Jiangsu Key Laboratory of Advanced Catalytic Materials and Technology, Analysis and Testing Center, NERC Biomass of Changzhou University, Changzhou 213164, China

**Keywords:** pterostilbene, synthesis, anti-BC activity, mitochondrial apoptosis

## Abstract

Pterostilbene has been found to be an active scaffold with anti-breast cancer (BC) action. In this study, fourteen pterostilbene-tethered analogues (**2A**–**2N**) were prepared and screened in vitro against MDA-MB-231 and MCF-7 cells. Meanwhile, their structures were characterized using ^1^H-NMR, ^13^C-NMR, and HRMS (ESI) spectroscopy techniques. Among them, analogue **2L** displayed the most potent anti-proliferation effect on MDA-MB-231 (IC_50_ = 10.39 μM) and MCF-7 cells (IC_50_ = 11.73 μM). Furthermore, the meaningful structure–activity relationships suggested that the introduction of a saturated six-membered nitrogen heterocyclic ring into the side chain favored anti-BC capacity. Biological observations indicated that **2L** could cause the typical morphological changes in apoptosis, namely an increase in reactive oxygen species level and a loss of mitochondrial membrane potential in BC cells. Importantly, **2L** could induce mitochondrial-mediated apoptosis by regulating the expression of caspase-related proteins. Consistent with the results of our in vitro study, **2L** apparently inhibited tumor growth in MDA-MB-231 xenograft mice without obvious toxicity. These findings revealed that **2L** is expected to be a promising anti-BC lead compound that merits further investigations.

## 1. Introduction

Breast cancer (BC) is one of the most prevalent malignancies and the second leading cause of cancer death among women worldwide [1,2]. Triple-negative breast cancer (TNBC), the most aggressive subtype of BC, features the absence of three classical membrane receptors (estrogen receptor, ER; progesterone receptor, PR; human epidermal growth factor receptor 2 positive, HER2) [3]. The limited therapeutic options for TNBC result in an undesirable prognosis and a high recurrence rate [4]. At present, conventional chemotherapy still occupies an important role in the combination therapy model for BC [5,6]. Unfortunately, its efficacy is hampered by several factors including drug resistance, severe side effects, and low selectivity [7,8]. To overcome these obstacles, some biological molecules, such as tubulin protein, cyclin-dependent kinase (CDK), epidermal growth factor (EGF), and Ras have been considered as appealing targets for antitumor drugs in the recent decades [9]. Therefore, the development of new chemotherapeutic agents for the treatment of BC is necessary.

**Figure 1:**
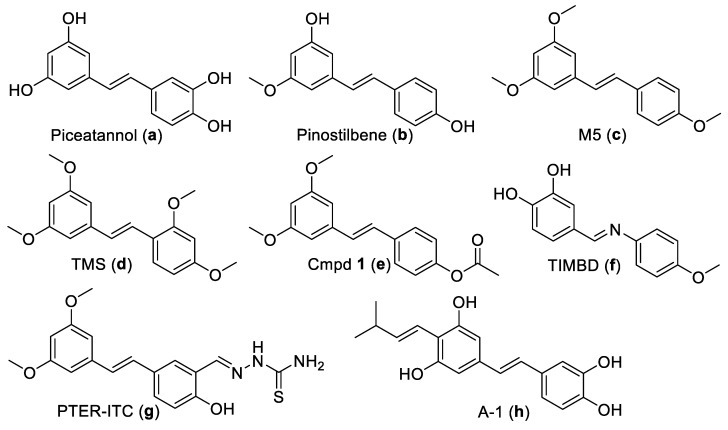
Chemical structures of pterostilbene-related derivatives (**a**–**h**) with anti-BC action.

Pterostilbene (3,5-dimethoxy-4′-hydroxy-trans-stilbene, PTE), a natural polyphenolic product, abundantly exists in *Pterocarpus marsupium*, blueberries, nuts, and *Vitis vinifera* leaves [10]. Structurally, PTE pertains to the homologue of resveratrol. Owing to the presence of a dimethoxy moiety, PTE is endowed with superior oral absorption, lipophilicity, and metabolic stability compared to resveratrol [11,12]. Notably, clinical trials have shown that PTE possesses a safe and tolerable profile for humans or animals even at heavy doses (250 mg/day) [13,14]. Similar to resveratrol, PTE exerts a broad spectrum of biological and pharmacological activities, including antioxidant, anti-inflammatory, anti-fungal, and anti-diabetic capacities [11,15,16,17]. In addition, PTE has been recognized as a potential antitumor agent against multiple types of cancers, including prostate, liver, lung, gastric, and colon cancer [18,19,20,21,22]. Preliminary investigations of its mechanisms have found that the anticancer behavior of PTE is mainly ascribable to the induction of apoptosis, cell cycle arrest, autophagy, and DNA damage, thus, inhibiting the growth and proliferation of tumor cells [23,24,25,26]. Of late, the important biological function of PTE against BC has gained extensive attention, especially its sensitivity to TNBC [27,28,29]. For this reason, several studies concerning the anti-BC capacity of some PTE-related derivatives (Figure 1, **a**–**h**) have been reported in the literature. These findings have implied PTE as a hallmark characteristic of an effective anti-BC lead drug molecule [27,30,31,32,33,34,35].

The discovery of anti-BC drug candidates has always been a part of our endeavors [36]. Meanwhile, encouraged by the above research background, we synthesized fourteen novel PTE-tethered derivatives (PTEs, **2A**–**2N**) in this study. Subsequently, all the target products were evaluated for their in vitro anti-proliferative activity against MDA-MB-231 and MCF-7 cells. At the same time, the structure–activity relationships (SARs) were preliminarily discussed. The most outstanding anti-BC candidate (**2L**) was screened to further investigate its effects on proliferation, reactive oxygen species, mitochondrial membrane potential, and apoptosis in vitro. Of note, the in vivo study suggested that **2L** exerted a significant anti-tumor potency in an MDA-MB-231 xenograft tumor model. These results indicated that **2L** has the potential to develop into a novel anti-BC lead compound.

## 2. Results and Discussion

### 2.1. Chemistry

**Scheme 1:**
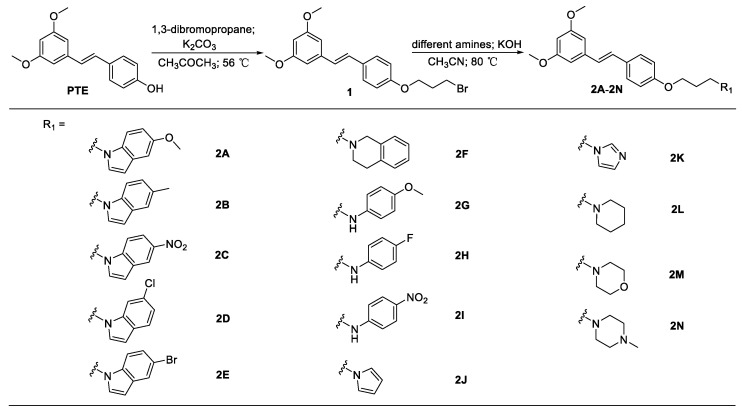
The synthesis of PTE-based derivatives (**2A**–**2N**).

Coinciding with the previous literature [18,19,20,21,22], our tentative bioassay screening found that PTE possessed anti-proliferation properties against MDA-MB-231 (TNBC, IC_50_ = 63.59 μM) and MCF-7 (ER^+^ BC, IC_50_ = 44.26 μM) cells. In this research, we aimed to obtain lead chemical entities with improved anti-BC activity through the structural optimization of PTE. As outlined in Scheme 1, a series of PTE analogues (**2A**–**2N**) were readily synthesized. PTE was reacted with 1,3-dibromopropane in the presence of potassium carbonate and acetone under reflux conditions to afford intermediate **1**. The title compounds (**2A**–**2N**) were prepared from **1** by nucleophilic substitution with appropriate aromatic amines, alicyclic amines, and anilines. All derivatives were purified through recrystallization or column chromatography with good yields, and their structures were confirmed using ^1^H-NMR, ^13^C-NMR, and HRMS (ESI) spectra.

### 2.2. Evaluation of Anti-BC Activity

The in vitro anti-proliferative activity of all synthesized analogues against MDA-MB-231 and MCF-7 cells was tested using the 3-(4,5-dimethylthiazol-2-yl)-3,5-phenytetrazoliumromide (MTT) method. Dox, a valuable anticancer drug, is highly effective in the treatment of various hematopoietic malignancies and solid tumors. Usually, Dox has been used as a positive control in many basic studies, especially drug screening assay [37,38]. As listed in Table 1, the IC_50_ values of most analogues (**1**, **2A**–**2J**) against BC cells were approximately greater than 100 μM, suggesting that they were almost inactive against BC cells at a 100 μM concentration. Only analogues **2K**–**2N** exerted the potential to moderate inhibition activity against BC cells with IC_50_ values ranging from 10.39 to 49.34 μM, which were obviously weaker than those of the positive control (Dox). Compared with the prototype drug (PTE), the derivatives **2K**, **2L**, and **2N** possessed excellent anti-BC activity, most impressively analogue **2L**. By contrast, analogue **2M** exhibited an inhibitory capacity against BC cells comparable to that of PTE. In the meantime, the cytotoxic activity of **2K**–**2N**, PTE, and Dox against MCF-10A (human breast epithelial cell line) was examined. Notably, the most active analogue **2L** showed a weak cytotoxicity against MCF-10A (IC_50_ = 68.87 μM) compared with analogues **2K** and **2N**. The preliminary SARs revealed that the implantation of aromatic moieties into the terminal alkyl fragment did not improve the anti-BC property (**2A**–**2I** vs. **1**, PTE). By contrast, the substitution of a saturated six-membered nitrogen heterocyclic ring on the side chain is extremely favorable for enhancing anti-BC efficacy (**2L**–**2N** vs. **1**, PTE, **2A**–**2J**). Based on the preceding analysis, analogue **2L** was selected as the most promising anti-BC agent and used in the follow-up study.

### 2.3. Effect of ***2L*** on BC Cells Proliferation

**Figure 2:**
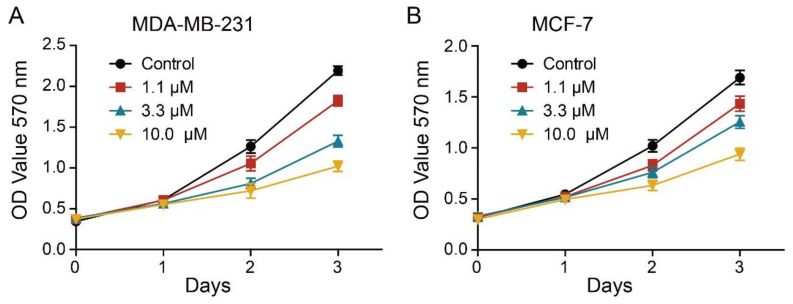
**2L** inhibited the proliferation of MDA-MB-231 (**A**) and MCF-7 (**B**) cells. The growth curve of BC cells treated with **2L** (1.1, 3.3, 10 μM) for 0, 1, 2, and 3 days. The control group was treated with vehicle.

To investigate the underlying mechanism of action of **2L**, we firstly inspected the effect of **2L** on the growth inhibition of MDA-MB-231 and MCF-7 cells by MTT assay. As depicted in Figure 2, the viability of MDA-MB-231 and MCF-7 cells decreased accordingly with the increase in **2L** concentration. These data suggested that **2L** significantly restrained the proliferation of BC cells in a concentration- and time-dependent manner. Compared to the control group, the viability of BC cells was not evidently influenced by **2L** within 24 h at the dosages of 1.1, 3.3, and 10 μM. Hence, these concentrations below the IC_50_ values of **2L** were chosen for the following experiments.

### 2.4. Effect of ***2L*** on Apoptosis of BC Cells

**Figure 3:**
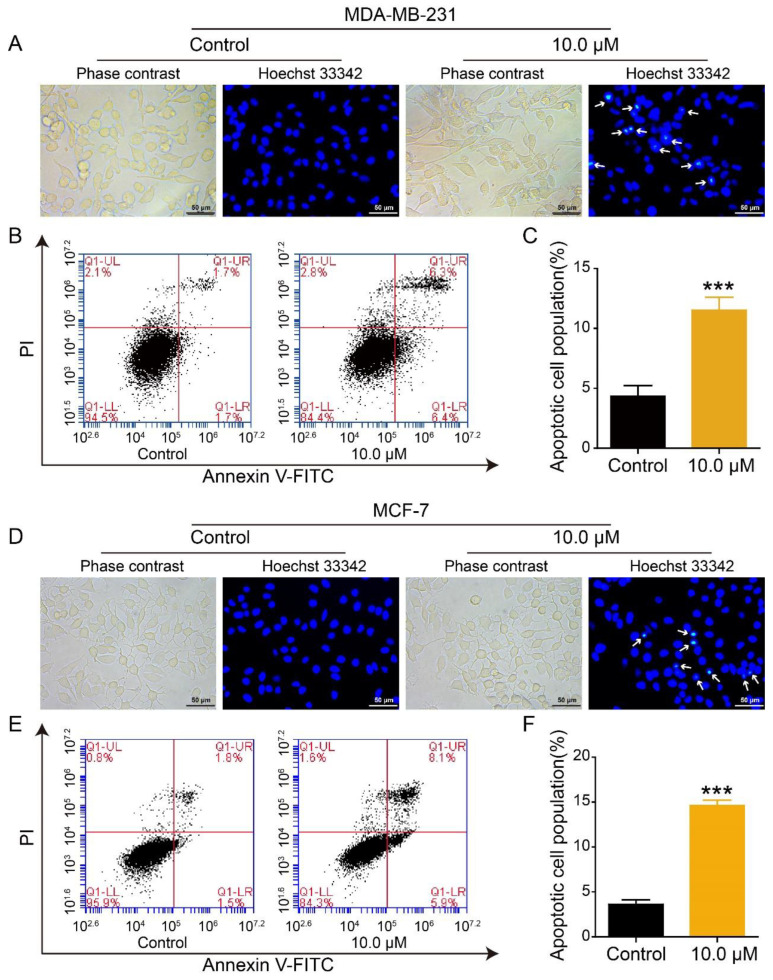
**2L** triggered apoptosis of BC cells. (**A**,**D**) Morphological alterations in **2L**-treated MDA-MB-231 and MCF-7 cells captured using fluorescence microscopy. Representative photographs were obtained (scale bar: 50 μm). The white arrows represent the nucleus of apoptotic BC cells. (**B**,**E**) Flow cytometric analysis of apoptosis of BC cells treated with **2L**. (**C**,**F**) The percentages of annexin V-positive BC cells. *** *p* < 0.001 vs. control.

To observe whether **2L** could cause the apoptosis of BC cells, Hoechst 33,342 staining was carried out. As shown in Figure 3A,D, the nuclei of BC cells in the control groups presented well-distributed fluorescence with a uniform circular and elliptical shape. However, the **2L**-treated groups exhibited the typical morphological changes in programmed apoptosis, including membrane blebbing, nuclear condensation, cell shrinkage, and apoptotic body formation. Subsequently, flow cytometric analysis of annexin V/PI was conducted. The apoptotic percentages in the control groups were about 3.4% (MDA-MB-231 cell) and 3.3% (MCF-7 cell), while the apoptosis proportion in the **2L**-treated groups increased to 12.7% and 14.0%, respectively (Figure 3B,E). At the same time, the results of the quantitative analysis showed that the total population of apoptotic BC cells in **2L**-treated groups was obviously elevated compared with the control groups (Figure 3C,F). These data indicated that analogue **2L** could induce apoptosis in BC cells.

### 2.5. Effect of ***2L*** on DNA Content of BC Cells

**Figure 4:**
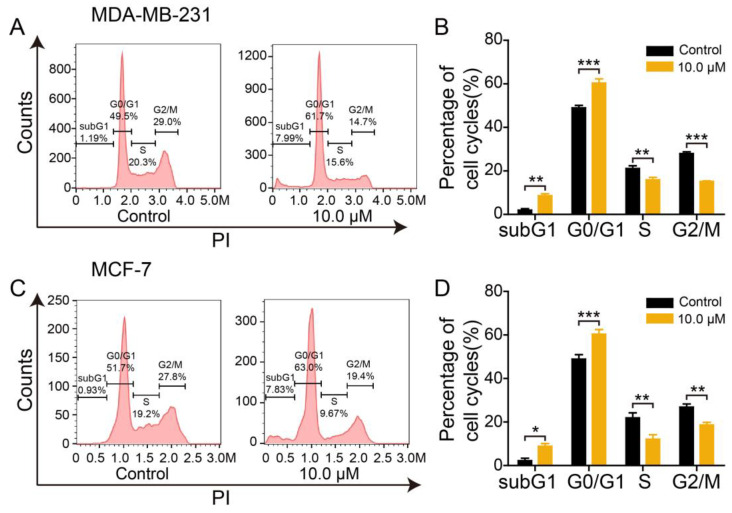
**2L** triggered cell cycle arrest of BC cells. (**A**,**C**) Cell cycle distribution of BC cells was determined using the flow cytometry. (**B**,**D**) Statistical analysis for the cell cycle distribution of BC cells. * *p* < 0.05, ** *p* < 0.01, *** *p* < 0.001 vs. control.

It is commonly acknowledged that intranucleosomal DNA fragmentation is a crucial hallmark of cell apoptosis [39]. Next, we assessed the influence of **2L** on the DNA content of PI-stained BC cells using flow cytometry. As shown in Figure 4A,C, the **2L**-treated groups exhibited a significant increase in the number of BC cells in the G0/G1 phase compared with the control groups. The relative proportion of MDA-MB-231 cells in the G0/G1 phase increased from 49.5% (control group) to 61.7% (**2L**-treated group). In turn, treatment with **2L** led to an apparent enhancement of the number of MCF-7 cells, from 51.7% to 63.0%. In particular, we found that **2L** could result in an increased percentage of subG1 apoptotic cells (up from 1.19% to 7.99% in MDA-MB-231 cells; from 0.93% to 7.83% in MCF-7 cells). An augmentation of DNA distribution in the SubG1 peak was representative of the occurrence of apoptosis in **2L**-exposed groups, which further confirmed that **2L** could induce the apoptosis of BC cells. In addition, the quantitative analysis of DNA distribution for the subG1, G0/G1, S, and G2/M phases appeared to show similar alterations (Figure 4B,D). These data indicated that **2L** could restrict the proliferation of BC cells through G0/G1 phase cell cycle arrest.

### 2.6. Effect of ***2L*** on Mitochondrial Membrane Potential

**Figure 5:**
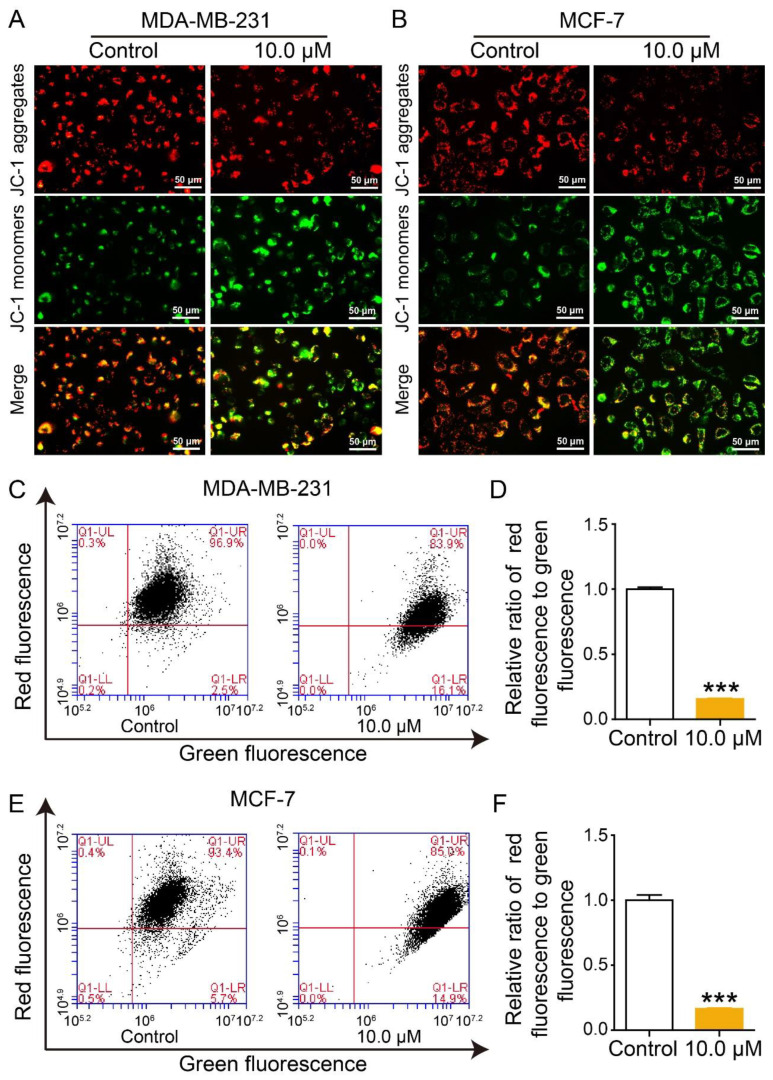
**2L** decreased MMP level in BC cells. (**A**,**B**) Fluorescence observation in MDA-MB-231 and MCF-7 cells under a fluorescence microscope (scale bar: 50 μm). (**C**,**E**) The variation in MMP level was measured using JC-1 assay kit. (**D**,**F**) Quantitative analysis of MMP in MDA-MB-231 and MCF-7 cells. *** *p* < 0.001 vs. control.

It is widely known that the pathways of cell apoptosis are mediated by mitochondria, endoplasmic reticulum stress, and death receptors [40]. Among them, intrinsic mitochondrial apoptosis has been considered as the canonical pathway of cell apoptosis [41]. In general, mitochondrial membrane potential (MMP) is a critical parameter for reflecting mitochondrial configuration and function [42]. To explore whether **2L**-induced apoptosis was involved with the disruption of mitochondrial membrane integrity, the variation of MMP (ΔΨm) was detected using fluorescent probe JC-1. Compared with the control group, the green fluorescence intensity in **2L**-treated BC cells was obviously enhanced, whereas the red fluorescence intensity was correspondingly attenuated (Figure 5A,B). Moreover, the results of flow cytometry manifested that the ΔΨ m level of MDA-MB-231 and MCF-7 cells sharply fell to 83.9% and 85.0%, respectively, after incubation with **2L** for 24 h (Figure 5C,E). In line with the above result, the quantitative analysis results showed that the proportion of red/green fluorescence in **2L**-administered groups drastically declined compared to control groups (Figure 5D,F). These observations indicated that **2L** could lead to a reduction in MMP, thus, triggering the early apoptosis of BC cells.

### 2.7. Effect of ***2L*** on Reactive Oxygen Species

**Figure 6:**
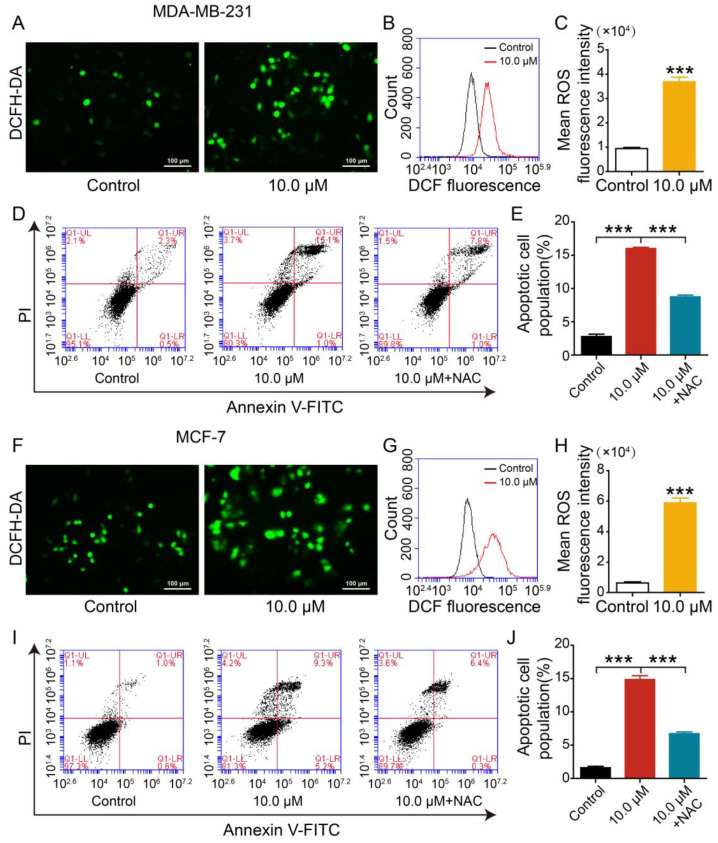
**2L** stimulated ROS production in MDA-MB-231 and MCF-7 cells and the role of ROS generation in **2L**-induced apoptosis. (**A**,**F**) The fluorescence distribution of **2L**-treated BC cells was observed and recorded (scale bar: 100 μm). (**B**,**G**) The variation in ROS level in BC cells was detected by flow cytometer. (**C**,**H**) Quantitative analysis of ROS in BC cells. (**D**,**I**) Apoptosis of cells was assessed by flow cytometry analyses after treatment with **2L** and **2L** + NAC. (**E**,**J**) Quantitative analysis of apoptosis rates by flow cytometry. *** *p* < 0.001 vs. control.

An increasing number of studies have emphasized the pivotal role of reactive oxygen species (ROS) homeostasis in cellular processes (e.g., proliferation, migration, and differentiation) and signaling cascades. Nevertheless, the abnormal elevation of ROS levels will initiate cell apoptosis and damage [43,44]. For this reason, we next determined if **2L** could stimulate the massive production of ROS using 2,7-dichloro-dihydrofluorescein diacetate (DCFH-DA) fluorescence staining. As shown in Figure 6A,F, the number of BC cells emitting green fluorescence was substantially increased in **2L**-administered groups, and the fluorescence intensity was also strengthened. Immediately after, flow cytometry was employed to quantitatively analyze the ROS production of **2L**-treated BC cells (Figure 6B,G). Compared with the control groups, the accumulation of ROS was significantly augmented in **2L**-treated groups. Likewise, the mean ROS-associated fluorescence intensity of **2L**-treated MDA-MB-231 and MCF-7 cells was greatly increased (Figure 6C,H). To further demonstrate the role of ROS in the **2L**-induced apoptosis, the cells were exposed to 5 mM NAC (a ROS scavenger) [45]. As depicted in Figure 6D,I, the apoptosis ratios (early and late stages) plummeted from 16.1% to 8.8% in MDA-MB-231 cells and from 14.5% to 6.7% in MCF-7 cells following **2L** + NAC treatment. Again, the quantitative analysis showed a similar tendency, indicating that NAC significantly attenuated **2L**-induced apoptosis (Figure 6E,J). Consequently, **2L** could trigger the apoptosis of BC cells via initiating the excessive generation of ROS.

### 2.8. Effect of ***2L*** on Apoptosis-Associated Proteins

**Figure 7:**
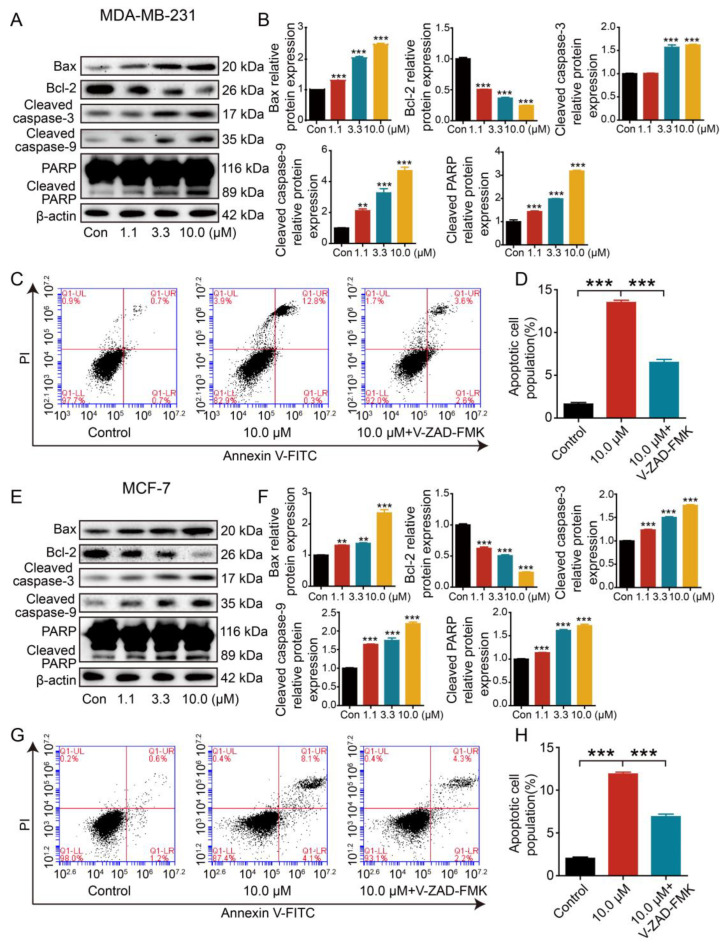
Effect of **2L** on apoptosis and apoptosis-associated proteins. (**A**,**E**) The expression levels of Bcl-2, Bax, cleaved caspase-3, cleaved caspase-9, PAPR, and cleaved PARP proteins in BC cells were inspected using Western blot. (**B**,**F**) The relative quantification of protein levels was analyzed using ImageJ software (V1.8.0). (**C**,**G**) The apoptosis rates were assessed by flow cytometry. (**D**,**H**) Quantitative analysis of apoptosis rates using flow cytometry. ** *p* < 0.01 and *** *p* < 0.001 vs. control.

Given that **2L** led to the production of intracellular ROS and loss of MMP, we speculated that **2L**-induced cell apoptosis was closely related to the mitochondria-dependent signaling pathway. Additionally, the mitochondrial pathway is modulated by the Bcl-2 family, caspase family, and oncogenes (e.g., C-myc, p53) [46]. In view of this, we continued to examine the expression of apoptosis-related proteins by Western blotting assay. As shown in Figure 7A,B,E,F, the expression of anti-apoptosis protein Bcl-2 was markedly downregulated in **2L**-treated cells, while the level of pro-apoptosis protein Bax was upregulated. Meanwhile, **2L** could increase the protein expression of cleaved caspase-9 and cleaved caspase-3. As we know, cleaved caspase-3 can activate its proteolytic activity, which further leads to the cleavage of poly(ADP-ribose) polymerase (PARP) [47]. Also, it was found that **2L** could effectively elevate the protein level of cleaved PARP. Generally, caspase-dependent apoptosis can be hindered by a classic caspase inhibitor Z-VAD-FMK [48]. To further determine whether **2L** induced caspase-dependent apoptosis, we carried out flow cytometry to analyze the apoptosis ratio of BC cells pretreated with Z-VAD-FMK (20 μM). As described in Figure 7C,G, the percentages of apoptotic cells in **2L**-alone groups were 13.1% (MDA-MB-231 cells) and 12.2% (MCF-7 cells), but the apoptosis ratios in **2L** + Z-VAD-FMK groups sharply dropped to 6.2% and 6.5%, respectively. At the same time, the results of quantitative analysis manifested that the apoptosis proportion of BC cells was significantly declined in the **2L** + Z-VAD-FMK groups compared with the **2L**-only groups (Figure 7D,H). These results indicated that the pretreatment of BC cells with Z-VAD-FMK could partly reverse the apoptosis of BC cells induced by **2L**. Overall, **2L** could activate apoptosis-related proteins, thereby inducing the apoptosis of BC cells through the mitochondrial pathway.

### 2.9. Evaluation of the Anti-Tumor Efficacy of ***2L*** In Vivo

**Figure 8:**
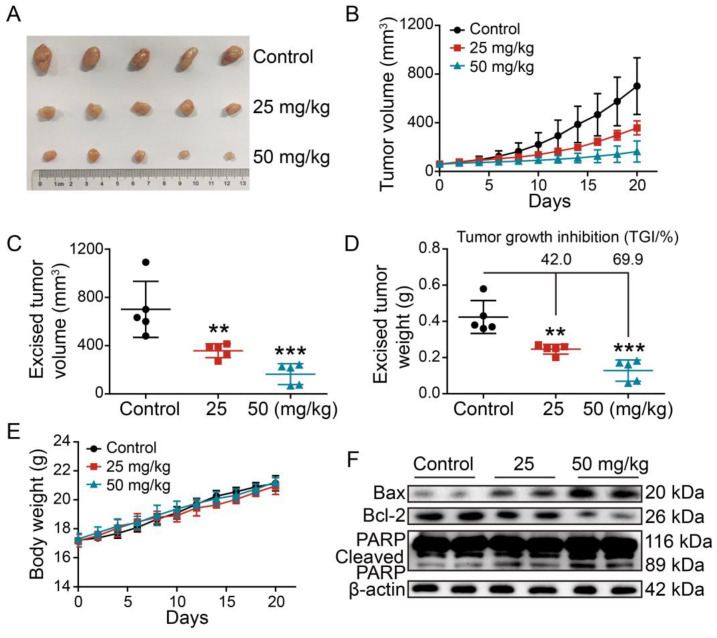
Anti-tumor efficacy of **2L** in the MDA-MB-231 xenograft tumor model. (**A**) Digital images of tumor tissues from different experimental groups. (**B**) The change curve of tumor volume. (**C**) The average tumor volume was measured after the mice were sacrificed. (**D**) The measurement of tumor weight and TGI. (**E**) The variation of body weight in each group. (**F**) Western blot analysis of Bcl-2, Bax, PARP, and cleaved PARP proteins in tumor tissues. ** *p* < 0.01, *** *p* < 0.001 vs. control group.

Considering the anti-tumor potency of **2L** in vitro, we next constructed the MDA-MB-231-derived xenograft mouse model to perform the in vivo anti-BC study. As illustrated in Figure 8A,B, **2L** effectively suppressed tumor size and growth in a dose-response curve. After the dissection of tumor tissues, the tumor volumes, weights, and tumor growth inhibition (TGI) ratios were calculated. The **2L**-treated groups showed an evident reduction in average tumor volume and weight. The TGI values of **2L** reached 42.0% and 69.9% at 25 and 50 mg/kg doses, respectively (Figure 8C,D). Again, the fluctuations in body weight in **2L**-administered groups were approximately the same as those in the control group, indicating that **2L** had no obvious toxicity in nude mice throughout the whole study (Figure 8E). Similar to the in vitro cell-based study, the apoptosis induced by **2L** was apparent in tumor tissues. Compared with the control group, the protein expression of Bax and cleaved PARP were noticeably increased in **2L**-treated groups, while the level of Bcl-2 protein was decreased (Figure 8F). These data suggested that **2L** remarkably inhibited the tumor proliferation of an MDA-MB-231 xenograft through the induction of cell apoptosis.

## 3. Materials and Methods

### 3.1. Chemistry

#### 3.1.1. General Procedures

All chemical reagents and solvents were purchased from commercial suppliers and used without further purification. Fresh THF and DCM were distilled from sodium wire and calcium hydride, respectively. The reactions were monitored by analytical thin layer chromatography (TLC) on Merck Silica Gel 60 F254 plates, which were visualized using UV radiation (254 nm) or further exposed to an ethanolic solution of concentrated sulfuric acid and anisaldehyde. The chromatographic purification was performed on silica gel (Qingdao Ocean Chemical Silicone Factory, Qingdao, China, 100–200 mesh, 200–300 mesh). The ^1^H and ^13^C NMR spectra were recorded on a Bruker AV-600 spectrometer using CDCl_3_ or DMSO-*d*_6_ as the solvent and tetramethylsilane as the internal standard. Signal multiplicity was designated as follows: s = singlet, d = doublet, t = triplet, q = quartet, quint = quintet, m = multiplet, dt = doublet of triplets, br = broad, and dd = doublet of doublets. The HR-ESI/MS measurements were taken with an Agilent G6230 TOF mass spectrometer.

#### 3.1.2. General Procedure for the Synthesis of the Title Compounds **2A**–**2N**

Here, 1,3-dibromopropane (1.5 equiv.) was slowly added to a mixed solution of PTE (1.0 equiv.) and anhydrous K_2_CO_3_ (2.5 equiv.) in acetone. After the almost complete consumption of PTE, the mixture was filtered. The filtrate was evaporated in vacuo and extracted three times with ethyl acetate (EtOAc). The combined organic phases were dried over anhydrous Na_2_SO_4_ and concentrated under reduced pressure. The resulting product was purified by flash chromatography column (petroleum ether/EtOAc = 50:1) to give intermediate **1**.

The appropriate aromatic amines, alicyclic amines, and anilines (1.8 equiv.) were gradually added to a stirred solution of **1** (1 equiv.) and KOH (3.0 equiv.) in MeCN for 6 h at 80 °C. After the completion of the reaction, the mixture was cooled to room temperature, filtered, and concentrated under vacuum. The crude product was purified by silica gel column chromatography to furnish the corresponding analogues **2A**–**2N**.

*(E)-1-(4-(3-bromopropoxy)styryl)-3,5-dimethoxybenzene* (**1**). White solid, 90% yield. ^1^H NMR (300 MHz, CDCl_3_) *δ* (ppm): 7.46 (d, *J* = 8.7 Hz, 2H), 7.06 (d, *J* = 16.2 Hz, 1H), 6.95 (d, *J* = 16.2 Hz, 1H), 6.92 (d, *J* = 8.7 Hz, 1H), 6.68 (d, *J* = 2.3 Hz, 2H), 6.41 (t, *J* = 2.2 Hz, 1H), 4.13 (t, *J* = 5.8 Hz, 2H), 3.85 (s, 6H), 3.63 (t, *J* = 6.4 Hz, 2H), 2.34 (quint, *J* = 6.1 Hz, 2H). ^13^C NMR (75 MHz, CDCl_3_) *δ* (ppm): 161.0 (2C), 158.5, 139.7, 130.2, 128.7, 127.9 (2C), 126.7, 114.8 (2C), 104.4 (2C), 99.7, 65.4, 55.4 (2C), 32.4, 30.1. (Supplementary Material Figures S1 and S2). HRMS (ESI) *m/z* calcd. for C_19_H_22_BrO_3_ [M + H]^+^ 377.0747, found 377.0752.

*(E)-1-(3-(4-(3,5-dimethoxystyryl)phenoxy)propyl)-5-methoxy-1H-indole* (**2A**). Eluent petroleum ether/EtOAc (50:1). Colorless oily, 94% yield. ^1^H NMR (300 MHz, CDCl_3_) *δ* (ppm): 7.61 (d, *J* = 8.7 Hz, 2H), 7.43 (d, *J* = 8.9 Hz, 1H), 7.29 (d, *J* = 2.4 Hz, 1H), 7.23 (d, *J* = 17.6 Hz, 1H), 7.21 (d, *J* = 3.1 Hz, 1H), 7.10 (d, *J* = 17.6 Hz, 1H), 7.06 (d, *J* = 8.7 Hz, 1H), 7.03 (d, *J* = 8.7 Hz, 2H), 6.85 (d, *J* = 2.2 Hz, 2H), 6.58 (m, 2H), 4.49 (t, *J* = 6.5 Hz, 2H), 4.03 (t, *J* = 6.5 Hz, 2H), 4.03 (s, 3H), 4.01 (s, 6H), 2.43 (quint, *J* = 6.1 Hz, 2H). ^13^C NMR (75 MHz, CDCl_3_) *δ* (ppm): 161.0 (2C), 158.5, 154.1, 139.7, 131.3, 130.2, 129.0, 128.7 (2C), 127.9 (2C), 126.7, 114.8 (2C), 112.0, 110.1, 104.4 (2C), 102.6, 100.9, 99.7, 64.3, 55.9, 55.4 (2C), 42.9, 29.9. (Supplementary Material Figures S3 and S4). HRMS (ESI) *m/z* calcd. for C_28_H_30_NO_4_ [M + H]^+^ 444.2169, found 444.2173.

*(E)-1-(3-(4-(3,5-dimethoxystyryl)phenoxy)propyl)-5-methoxy-1H-indole* (**2B**). Eluent petroleum ether/EtOAc (30:1). Colorless oily, 96% yield. ^1^H NMR (300 MHz, CDCl_3_) *δ* (ppm): 7.50 (m, 3H), 7.31 (d, *J* = 8.4 Hz, 1H), 7.11 (d, *J* = 16.4 Hz, 1H), 7.08 (m, 2H), 6.97 (d, *J* = 16.4 Hz, 1H), 6.91 (d, *J* = 8.7 Hz, 2H), 6.72 (d, *J* = 2.2 Hz, 2H), 6.45 (m, 2H), 4.37 (t, *J* = 6.5 Hz, 2H), 3.89 (t, *J* = 6.5 Hz, 2H), 3.88 (s, 6H), 2.51 (s, 3H), 2.30 (quint, *J* = 6.1 Hz, 2H). ^13^C NMR (75 MHz, CDCl_3_) *δ* (ppm): 161.1 (2C), 158.6, 139.7, 134.4, 130.2, 129.0, 128.8, 128.6, 128.3, 127.9 (2C), 126.7, 123.2, 120.7, 114.8 (2C), 109.1, 104.4 (2C), 100.8, 99.7, 64.4, 55.4 (2C), 42.8, 29.9, 21.5. (Supplementary Material Figures S5 and S6). HRMS (ESI) *m/z* calcd. for C_28_H_30_NO_3_ [M + H]^+^ 428.2220, found 428.2227.

*(E)-1-(3-(4-(3,5-dimethoxystyryl)phenoxy)propyl)-5-nitro-1H-indole* (**2C**). Eluent petroleum ether/EtOAc (15:1). Yellow solid, 92% yield. ^1^H NMR (300 MHz, CDCl_3_) *δ* (ppm): 8.57 (d, *J* = 2.2 Hz, 1H), 8.07 (dd, *J* = 9.2, 2.2 Hz, 1H), 7.44 (d, *J* = 8.3 Hz, 2H), 7.37 (d, *J* = 9.1 Hz, 1H), 7.24 (d, *J* = 3.3 Hz, 1H), 7.04 (d, *J* = 16.2 Hz, 1H), 6.91 (d, *J* = 16.2 Hz, 1H), 6.86 (d, *J* = 8.3 Hz, 2H), 6.66 (m, 3H), 6.39 (t, *J* = 2.2 Hz, 1H), 4.43 (t, *J* = 6.6 Hz, 2H), 3.89 (t, *J* = 5.6 Hz, 2H), 3.83 (s, 6H), 2.31 (quint, *J* = 6.1 Hz, 2H). ^13^C NMR (75 MHz, CDCl_3_) *δ* (ppm): 161.0 (2C), 158.2, 141.6, 139.6, 138.9, 131.3, 130.4, 128.5, 127.9 (2C), 127.8, 126.9, 118.3, 117.3, 114.7, 109.2, 104.4 (2C), 104.2, 99.7, 64.0, 55.4 (2C), 43.3, 29.8, 29.7. (Supplementary Material Figures S7 and S8). HRMS (ESI) *m/z* calcd. for C_27_H_27_N_2_O_5_ [M + H]^+^ 459.1914, found 459.1909.

*(E)-6-chloro-1-(3-(4-(3,5-dimethoxystyryl)phenoxy)propyl)-1H-indole* (**2D**). Eluent petroleum ether/EtOAc (20:1). Light yellow solid, 90% yield. ^1^H NMR (300 MHz, CDCl_3_) *δ* (ppm): 7.54 (d, *J* = 8.4 Hz, 1H), 7.46 (d, *J* = 8.7 Hz, 2H), 7.38 (m, 1H), 7.09 (d, *J* = 8.7 Hz, 1H), 7.08 (d, *J* = 16.6 Hz, 1H), 7.07 (d, *J* = 1.8 Hz, 1H), 6.93 (d, *J* = 16.6 Hz, 1H), 6.89 (d, *J* = 8.8 Hz, 2H), 6.69 (d, *J* = 2.3 Hz, 2H), 6.47 (dd, *J* = 3.1, 0.9 Hz, 1H), 6.42 (t, *J* = 2.2 Hz, 1H), 4.33 (t, *J* = 6.6 Hz, 2H), 3.87 (d, *J* = 6.6 Hz, 2H), 3.85 (s, 6H), 2.26 (quint, *J* = 6.2 Hz, 2H). ^13^C NMR (75 MHz, CDCl_3_) *δ* (ppm): 161.0 (2C), 158.4, 139.7, 136.4, 130.3, 128.9, 128.7, 127.9 (2C), 127.6, 127.2, 126.8, 121.9, 120.1, 114.7 (2C), 109.5, 104.4 (2C), 101.6, 99.7, 64.2, 55.4 (2C), 42.9, 29.7. (Supplementary Material Figures S9 and S10). HRMS (ESI) *m/z* calcd. for C_27_H_27_ClNO_3_ [M + H]^+^ 448.1674, found 448.1680.

*(E)-5-bromo-1-(3-(4-(3,5-dimethoxystyryl)phenoxy)propyl)-1H-indole* (**2E**). Eluent petroleum ether/EtOAc (20:1). White solid, 88% yield. ^1^H NMR (300 MHz, CDCl_3_) *δ* (ppm): 7.82 (d, *J* = 1.7 Hz, 1H), 7.50 (d, *J* = 8.7 Hz, 2H), 7.30 (m, 2H), 7.13 (d, *J* = 3.4 Hz, 1H), 7.12 (d, *J* = 16.3 Hz, 1H), 6.98 (d, *J* = 16.3 Hz, 1H), 6.92 (d, *J* = 8.7 Hz, 2H), 6.73 (d, *J* = 2.2 Hz, 2H), 6.47 (m, 2H), 4.39 (t, *J* = 6.6 Hz, 2H), 3.91 (t, *J* = 6.6 Hz, 2H), 3.90 (s, 6H), 2.31 (quint, *J* = 6.2 Hz, 2H). ^13^C NMR (75 MHz, CDCl_3_) *δ* (ppm): 161.0 (2C), 158.4, 139.7, 134.7, 130.32, 130.27, 129.3, 128.7, 127.9 (2C), 126.8, 124.4, 123.5, 114.7 (2C), 112.7, 110.8, 104.4 (2C), 101.0, 99.7, 64.1, 55.4 (2C), 42.9, 29.8. (Supplementary Material Figures S11 and S12). HRMS (ESI) *m/z* calcd. for C_27_H_27_BrNO_3_ [M + H]^+^ 492.1169, found 492.1176.

*(E)-2-(3-(4-(3,5-dimethoxystyryl)phenoxy)propyl)-1,2,3,4-tetrahydroisoquinoline* (**2F**). Eluent petroleum ether/EtOAc (10:1). White solid, 94% yield. ^1^H NMR (300 MHz, CDCl_3_) *δ* (ppm): 7.45 (d, *J* = 8.7 Hz, 2H), 7.16–7.12 (m, 3H), 7.08–7.03 (m, 2H), 6.94–6.89 (m, 3H), 6.67 (d, *J* = 2.3 Hz, 2H), 6.39 (t, *J* = 2.2 Hz, 1H), 4.10 (t, *J* = 6.4 Hz, 2H), 3.84 (s, 6H), 3.68 (s, 2H), 2.94 (t, *J* = 5.9 Hz, 2H), 2.78 (t, *J* = 5.9 Hz, 2H), 2.72 (t, *J* = 7.2 Hz, 2H), 2.10 (quint, *J* = 7.5 Hz, 2H). ^13^C NMR (75 MHz, CDCl_3_) *δ* (ppm): 161.0 (2C), 158.9, 139.7, 134.8, 134.3, 129.8, 128.8, 128.7, 127.8 (2C), 126.6, 126.5, 126.2, 125.6, 114.8 (2C), 104.3 (2C), 99.6, 66.3, 56.3, 55.4 (2C), 55.0, 51.0, 29.2, 27.1. (Supplementary Material Figures S13 and S14). HRMS (ESI) *m/z* calcd. for C_28_H_32_NO_3_ [M + H]^+^ 430.2382, found 430.2390.

*(E)-N-(3-(4-(3,5-dimethoxystyryl)phenoxy)propyl)-4-methoxyaniline* (**2G**). Eluent petroleum ether/EtOAc (25:1). Light yellow solid, 83% yield. ^1^H NMR (300 MHz, CDCl_3_) *δ* (ppm): 7.45 (d, *J* = 8.7 Hz, 2H), 7.05 (d, *J* = 16.3 Hz, 1H), 6.90 (d, *J* = 8.8 Hz, 2H), 6.88 (d, *J* = 16.3 Hz, 1H), 6.79 (d, *J* = 9.0 Hz, 2H), 6.66 (d, *J* = 2.4 Hz, 2H), 6.61 (d, *J* = 8.9 Hz, 2H), 6.38 (t, *J* = 2.2 Hz, 1H), 4.11 (t, *J* = 5.9 Hz, 2H), 3.83 (s, 6H), 3.75 (s, 3H), 3.32 (t, *J* = 6.7 Hz, 2H), 2.10 (quint, *J* = 6.2 Hz, 2H). ^13^C NMR (75 MHz, CDCl_3_) *δ* (ppm): 161.0 (2C), 158.6, 152.1, 142.5, 139.7, 130.0, 128.7, 127.8 (2C), 126.6, 115.0 (2C), 114.7 (2C), 114.2 (2C), 104.3 (2C), 99.6, 66.1, 55.8, 55.4 (2C), 42.2, 29.2. (Supplementary Material Figures S15 and S16). HRMS (ESI) *m/z* calcd. for C_26_H_30_NO_4_ [M + H]^+^ 420.2175, found 420.2188.

*(E)-N-(3-(4-(3,5-dimethoxystyryl)phenoxy)propyl)-4-fluoroaniline* (**2H**). Eluent petroleum ether/EtOAc (20:1). Light yellow oily, 81% yield. ^1^H NMR (300 MHz, CDCl_3_) *δ* (ppm): 7.45 (d, *J* = 8.7 Hz, 2H), 7.05 (d, *J* = 16.3 Hz, 1H), 6.94–6.87 (m, 5H), 6.66 (d, *J* = 2.3 Hz, 2H), 6.57 (d, *J* = 9.0 Hz, 1H), 6.56 (d, *J* = 9.0 Hz, 1H), 6.39 (t, *J* = 2.2 Hz, 1H), 4.11 (t, *J* = 5.8 Hz, 2H), 3.84 (s, 6H), 3.32 (t, *J* = 6.6 Hz, 2H), 2.10 (quint, *J* = 6.6 Hz, 2H). ^13^C NMR (75 MHz, CDCl_3_) *δ* (ppm): 161.0 (2C), 158.6, 157.4, 154.2, 144.61, 144.59, 139.7, 130.1, 128.7, 127.9 (2C), 126.7, 115.8, 115.5, 114.7 (2C), 113.6, 113.5, 104.4 (2C), 99.6, 66.1, 55.4 (2C), 41.9, 29.0. (Supplementary Material Figures S17 and S18). HRMS (ESI) *m/z* calcd. for C_25_H_27_FNO_3_ [M + H]^+^ 408.1975, found 408.1986.

*(E)-N-(3-(4-(3,5-dimethoxystyryl)phenoxy)propyl)-4-nitroaniline* (**2I**). Eluent petroleum ether/EtOAc (15:1). Light yellow solid, 87% yield. ^1^H NMR (300 MHz, CDCl_3_) *δ* (ppm): 8.07 (d, *J* = 9.2 Hz, 2H), 7.45 (d, *J* = 8.7 Hz, 2H), 7.04 (d, *J* = 16.3 Hz, 1H), 6.91 (d, *J* = 16.3 Hz, 1H), 6.89 (d, *J* = 8.6 Hz, 2H), 6.66 (d, *J* = 2.2 Hz, 2H), 6.54 (d, *J* = 9.2 Hz, 2H), 6.39 (t, *J* = 2.2 Hz, 1H), 4.11 (t, *J* = 5.6 Hz, 2H), 3.83 (s, 6H), 3.45 (q, *J* = 6.3 Hz, 2H), 2.13 (quint, *J* = 6.0 Hz, 2H). ^13^C NMR (75 MHz, CDCl_3_) *δ* (ppm): 161.0 (2C), 158.3, 153.3, 139.6, 137.9, 130.4, 128.6, 127.9 (2C), 126.9, 126.5 (2C), 114.6 (2C), 111.0 (2C), 104.4 (2C), 99.7, 65.9, 55.4 (2C), 41.0, 28.6. (Supplementary Material Figures S19 and S20). HRMS (ESI) *m/z* calcd. for C_25_H_27_N_2_O_5_ [M + H]^+^ 435.1914, found 435.1920.

*(E)-1-(3-(4-(3,5-dimethoxystyryl)phenoxy)propyl)-1H-pyrrole* (**2J**). Eluent petroleum ether/EtOAc (20:1). White crystal, 95% yield. ^1^H NMR (300 MHz, CDCl_3_) *δ* (ppm): 7.46 (d, *J* = 8.7 Hz, 2H), 7.07 (d, *J* = 16.2 Hz, 1H), 6.94 (d, *J* = 16.2 Hz, 1H), 6.90 (d, *J* = 8.7 Hz, 2H), 6.68 (d, *J* = 2.1 Hz, 4H), 6.41 (t, *J* = 2.3 Hz, 1H), 6.18 (t, *J* = 2.1 Hz, 2H), 4.14 (t, *J* = 6.7 Hz, 2H), 3.92 (t, *J* = 5.8 Hz, 2H), 3.92 (s, 6H), 2.23 (quint, *J* = 6.2 Hz, 2H). ^13^C NMR (75 MHz, CDCl_3_) *δ* (ppm): 161.0 (2C), 158.6, 139.7, 130.2, 128.7, 127.9 (2C), 126.7, 120.8 (2C), 114.7 (2C), 108.2 (2C), 104.4 (2C), 99.7, 64.3, 55.4 (2C), 46.0, 31.3. (Supplementary Materials Figures S21 and S22). HRMS (ESI) *m/z* calcd. for C_23_H_26_NO_3_ [M + H]^+^ 364.1907, found 364.1913.

*(E)-1-(3-(4-(3,5-dimethoxystyryl)phenoxy)propyl)-1H-imidazole* (**2K**). Eluent petroleum ether/EtOAc (1:2). White solid, 91% yield. ^1^H NMR (300 MHz, CDCl_3_) *δ* (ppm): 7.46 (s, 1H), 7.42 (d, *J* = 8.7 Hz, 2H), 7.05–7.00 (m, 2H), 6.92–6.83 (m, 4H), 6.64 (d, *J* = 2.2 Hz, 2H), 6.37 (t, *J* = 2.2 Hz, 1H), 4.16 (t, *J* = 6.8 Hz, 2H), 3.89 (t, *J* = 5.7 Hz, 2H), 3.81 (s, 6H), 2.20 (quint, *J* = 6.2 Hz, 2H). ^13^C NMR (75 MHz, CDCl_3_) *δ* (ppm): 161.0 (2C), 158.2, 139.6, 137.3, 130.4, 129.6, 128.6, 127.9 (2C), 126.8, 119.0, 114.7 (2C), 104.4 (2C), 99.7, 63.8, 55.4 (2C), 43.4, 30.8. (Supplementary Material Figures S23 and S24). HRMS (ESI) *m/z* calcd. for C_22_H_25_N_2_O_3_ [M + H]^+^ 365.1860, found 365.1853.

*(E)-1-(3-(4-(3,5-dimethoxystyryl)phenoxy)propyl)piperidine* (**2L**). Eluent petroleum ether/EtOAc (1:3). White solid, 93% yield. ^1^H NMR (300 MHz, CDCl_3_) *δ* (ppm): 7.43 (d, *J* = 8.7 Hz, 2H), 7.04 (d, *J* = 16.3 Hz, 1H), 6.90 (d, *J* = 16.3 Hz, 1H), 6.89 (d, *J* = 8.8 Hz, 2H), 6.65 (d, *J* = 2.2 Hz, 2H), 6.37 (t, *J* = 2.2 Hz, 1H), 4.02 (t, *J* = 6.4 Hz, 2H), 3.82 (s, 6H), 2.54–2.34 (m, 6H), 1.98 (m, 2H), 1.60 (m, 4H), 1.45 (m, 2H). ^13^C NMR (75 MHz, CDCl_3_) *δ* (ppm): 161.0 (2C), 158.9, 139.7, 129.8, 128.8, 127.8 (2C), 126.4, 114.7 (2C), 104.3 (2C), 99.6, 66.6, 56.0, 55.4 (2C), 54.7 (2C), 26.8, 26.0 (2C), 24.4. (Supplementary Materials Figures S25 and S26). HRMS (ESI) *m/z* calcd. for C_24_H_32_NO_3_ [M + H]^+^ 382.2382, found 382.2377.

*(E)-4-(3-(4-(3,5-dimethoxystyryl)phenoxy)propyl)morpholine* (**2M**). Eluent petroleum ether/EtOAc (1:1). White solid, 89% yield. ^1^H NMR (300 MHz, CDCl_3_) *δ* (ppm): 7.43 (d, *J* = 8.7 Hz, 2H), 7.04 (d, *J* = 16.2 Hz, 1H), 6.90 (d, *J* = 16.2 Hz, 1H), 6.89 (d, *J* = 8.8 Hz, 2H), 6.65 (d, *J* = 2.2 Hz, 2H), 6.38 (t, *J* = 2.2 Hz, 1H), 4.03 (t, *J* = 6.3 Hz, 2H), 3.82 (s, 6H), 3.75 (t, *J* = 4.5 Hz, 4H), 2.56–2.40 (m, 6H), 1.97 (quint, *J* = 6.6 Hz, 2H). ^13^C NMR (75 MHz, CDCl_3_) *δ* (ppm): 161.0 (2C), 158.8, 139.7, 129.9, 128.7, 127.8 (2C), 126.5, 114.7 (2C), 104.3 (2C), 99.6, 67.0 (2C), 66.2, 55.6, 55.4 (2C), 53.8 (2C), 26.5. (Supplementary Materials Figures S27 and S28). HRMS (ESI) *m/z* calcd. for C_23_H_30_NO_4_ [M + H]^+^ 384.2175, found 384.2183.

*(E)-1-(3-(4-(3,5-dimethoxystyryl)phenoxy)propyl)-4-methylpiperazine* (**2N**). Eluent dichloromethane/methanol (30:1). White solid, 86% yield. ^1^H NMR (300 MHz, DMSO-*d*_6_) *δ* (ppm): 7.52 (d, *J* = 8.3 Hz, 2H), 7.21 (d, *J* = 16.3 Hz, 1H), 7.02 (d, *J* = 16.3 Hz, 1H), 6.93 (d, *J* = 8.3 Hz, 2H), 6.74 (d, *J* = 2.2 Hz, 2H), 6.39 (t, *J* = 2.2 Hz, 1H), 4.01 (t, *J* = 6.4 Hz, 2H), 3.77 (s, 6H), 2.51 (m, 2H), 2.45–2.24 (m, 8H), 2.17 (s, 3H), 1.86 (quint, *J* = 6.8 Hz, 2H). ^13^C NMR (75 MHz, DMSO-*d*_6_) *δ* (ppm): 161.1 (2C), 158.9, 139.9, 129.9, 129.0, 128.3 (2C), 126.5, 115.1 (2C), 104.6 (2C), 99.9, 66.3, 55.6 (2C), 55.1 (2C), 54.8, 53.0 (2C), 46.1, 26.6. (Supplementary Material Figures S29 and S30). HRMS (ESI) *m/z* calcd. for C_24_H_33_N_2_O_3_ [M + H]^+^ 397.2486, found 397.2474.

### 3.2. Biological Evaluation

#### 3.2.1. Cell Culture and Cell Proliferation Assay

The human breast cancer cell lines MDA-MB-231 and MCF-7 were purchased from the Chinese Academy of Science and cultured in Dulbecco’s modified Eagle’s medium (DMEM, Gibco, New York, NY, USA) containing 10% fetal bovine serum (FBS), 2 mM L-glutamine, 100 U/mL penicillin, and 100 μg/mL streptomycin. Then, the cells were maintained in a humidified incubator at 37 °C in the presence of 5% CO_2_.

The MTT assay was used to test the effect of PTEs on cell viability. The MDA-MB-231 (3 × 10^3^ cells/well) and MCF-7 (4 × 10^3^ cells/well) cells were seeded in a 96-well plate and cultured at 37 °C in a 5% CO_2_ incubator overnight. Afterwards, the medium was replaced with different concentrations of the title compounds (1, 3, 10, 30, and 100 μM). After incubation for 48 h, 10 μL of MTT solution (5 mg/mL) was added to each well and incubated for another 4 h. The optical density (OD) of the samples was measured using a microplate reader (Biotek, Burlington, VT, USA) at 570 nm. The inhibition rate (%) = [A570 (control) − A570 (compound)]/A570 (control) × 100%. The IC_50_ values were calculated using SPSS software version 17.0 (SPSS Inc., Chicago, IL, USA).

#### 3.2.2. Hoechst Staining

MDA-MB-231 and MCF-7 cells were inoculated in 24-well plates with a density of 6 × 10^4^ cells/well. After treatment with **2L** (0, 10 μM) for 24 h, the cells were washed three times with phosphate-buffered saline (PBS), followed by staining with Hoechst 33,342 in the dark for 5 min. The nuclear images were observed with a DMI3000B fluorescence microscope (Leica, Solms, Germany), and the changes in nuclear morphology were captured.

#### 3.2.3. Cell Apoptosis Analysis

MDA-MB-231 and MCF-7 cells (3 × 10^5^ cells/well) were seeded into 6-well plates. After that, the cells were treated with **2L** (0, 10 μM) for 24 h. Cells were harvested, washed repeatedly with cold phosphate-buffered saline (PBS), and resuspended in 195 μL of binding buffer. Then, 4 μL annexin V-FITC reagent (Beyotime) and 8 μL propidium iodide (PI, Beyotime) were added to the samples and gently mixed. After incubation for 15 min in the dark at room temperature, the stained cells were immediately detected by a FACScan flow cytometer (BD Biosciences, San Jose, CA, USA) and analyzed with the annexin V-FITC/PI apoptosis method.

#### 3.2.4. Mitochondrial Membrane Potential (MMP) Assay

The MMP was assayed using a JC-1 assay kit (Beyotime, Shanghai, China) according to the manufacturer’s instructions. In brief, MDA-MB-231 and MCF-7 cells were plated in 24-well plates at a density of 6 × 10^4^ cells per well. After intervention with **2L** (0, 10 μM) for 24 h, the cells were washed once with PBS. Cell culture solution (0.5 mL) and JC-1 working solution (0.5 mL) were successively added, mixed well, and incubated at 37 °C for 20 min in the dark. After washing twice with 1 × JC-1 staining buffer, the cells were resuspended in fresh culture medium. The graphical results were observed under a fluorescence microscope.

MDA-MB-231 and MCF-7 cells were incubated in 6-well plates at a density of 3 × 10^5^ cells per dish and exposed to **2L** for 24 h. Cell culture media (1 mL) and JC-1 staining working solution (1 mL) were added, thoroughly mixed, and incubated at 37 °C for 20 min. After washing twice with 1 × JC-1 staining buffer, the cells were collected and resuspended in 500 μL JC-1 staining buffer and then instantly analyzed using a flow cytometry.

#### 3.2.5. Measurement of Intracellular ROS Levels

The Reactive Oxygen Species Assay Kit was used to measure intracellular ROS levels. MDA-MB-231 and MCF-7 cells (6 × 10^4^ cells/well) were seeded onto 24-well plates and treated with **2L** (0, 10 μM) for 24 h. Subsequently, the cells were washed with PBS and stained with dichlorodihydrofluorescein diacetate (DCFH-DA, 10 μM) at 37 °C for 20 min in the dark. Following three washes with serum-free DMEM, the fluorescence alteration was photographed using a fluorescence microscope.

MDA-MB-231 and MCF-7 cells were plated in 6-well plates with a density of 3 × 10^5^ cells/well. After exposure to **2L** for 24 h, the cells were incubated with 10 μM DCFH-DA at 37 °C for 20 min in the dark. Later, the cells were slightly washed three times with serum-free DMEM and centrifuged (1000 rpm, 5 min). The collected cells were resuspended in PBS and loaded into a flow cytometer.

#### 3.2.6. In Vivo Models

BALB/c nude mice (female, 6–7 weeks) were purchased from Changsheng Biotechnology Co., Ltd. (Liaoning, China). MDA-MB-231 cells (5 × 10^6^ cells/mouse) were injected subcutaneously into the right back of the nude mice. When the tumor volume reached approximately 55 mm^3^, the female mice were randomly divided into two treatment groups (25, 50 mg/kg) and a vehicle group (equal volume of normal saline) with five mice in each group. Throughout the experiment, **2L** and saline were intraperitoneally injected every other day for 10 treatments. At the same time, the changes in tumor volume and body weight of the nude mice were tracked and recorded. The tumor volumes were assessed using the formula: V (mm^3^) = (length × width^2^)/2. At the end of the experimental period, tumor tissues were harvested, weighed, and collected. The TGI rate was calculated by the average tumor weight of the **2L**-treated and control groups.

#### 3.2.7. Western Blot Assay

MDA-MB-231 and MCF-7 cells were cultured in a 6-well plate with the density of 3 × 10^5^ cells/well and treated with different concentrations of **2L** (0, 1.1, 3.3, 10 μM) for 24 h. The cells were lysed in radioimmunoprecipitation assay (RIPA) buffer (Beyotime, Shanghai, China) containing 1% protease inhibitor cocktail and 1 mM phenylmethylsulfonyl fluoride (PMSF, Sigma-Aldrich, St. Louis, MO, USA). After centrifugation, the supernatant was collected and quantified by BCA assay (Beyotime). Lysates were mixed with the loading buffer (5 ×) and denatured by boiling for 10 min. Then, the equivalent samples were separated via 10–15% sodium dodecyl–sulfate polyacrylamide gel electrophoresis (SDS-PAGE), transferred to 0.45 µm immunoblot poly(vinylidene fluoride) (PVDF) membranes (Merck Millipore, Darmstadt, Germany) at 4 °C, and blocked in 5% skimmed milk in TBST for 1 h at room temperature. The membranes were incubated overnight at 4 °C with specific primary antibodies. After washing three times with TBST, the PVDF membranes were incubated with horseradish peroxidase (HRP)-conjugated secondary antibodies at rt for 1 h. Lastly, the resulting bands were visualized by a Bio-Rad ChemiDoc imaging system (Thermo Fisher Scientific, Waltham, MA, USA) using enhanced chemiluminescence reagents (Millipore, Billerica, MA, USA). The details of the corresponding primary antibodies were as follows: Bax (2772; Cell Signaling Technology), Bcl-2 (3498; Cell Signaling Technology), cleaved caspase-3 (9661; Cell Signaling Technology), cleaved caspase-9 (9505; Cell Signaling Technology), and PARP (9542; Cell Signaling Technology). The HRP-secondary antibodies were also obtained from Cell Signaling Technology (Boston, MA, USA).

#### 3.2.8. Statistical Analysis

All data were presented as mean ± SEM from three independent experiments and statistically analyzed using Student’s *t* test. A one-way analysis of variance (ANOVA) and least significant difference (LSD) post hoc test were used to calculate group differences (SPSS version 17 software). * *p* < 0.05, ** *p* < 0.01, *** *p* < 0.001, and ns (no significant) were considered as the statistical significance.

## 4. Conclusions

In conclusion, fourteen pterostilbene-tethered analogues were smoothly achieved and evaluated for their anti-BC efficacy. The primary structure–activity relationship demonstrated that the existence of a saturated nitrogen heterocycle on the side chain was beneficial for anti-BC behavior. Among all of the analogues, **2L** exhibited the best inhibitory potency on MDA-MB-231 and MCF-7 cells. Biological studies revealed that **2L** could cause the morphological alteration, aberrant ROS generation, MMP depolarization, and the induction of apoptosis-related proteins, thus, triggering the mitochondrial-dependent apoptotic pathway. More importantly, **2L** could evidently restrain the growth of an MDA-MB-231 tumor xenograft via inducing apoptosis. Collectively, these results implied the potential of **2L** as an attractive anti-BC drug candidate.

## Figures and Tables

**Table 1:** The in vitro anti-BC activity of **1** and **2A**–**2N** ^a^.

Compounds	BC Cell Lines (IC_50_: μM)		MCF-10A (IC_50_: μM)
	MDA-MB-231	MCF-7	
**2K**	18.65 ± 0.71 ***	21.01 ± 0.56 ***	49.03 ± 1.31
**2L**	10.39 ± 0.40 ***	11.73 ± 0.55 ***	68.87 ± 0.93
**2M**	49.34 ± 2.48 *	42.03 ± 0.79	70.39 ± 0.43
**2N**	17.70 ± 0.81 ***	18.06 ± 0.55 ***	37.33 ± 0.96
PTE	63.59 ± 0.40	44.26 ± 0.48	>100
Dox ^b^	2.30 ± 0.32	3.57 ± 0.41	2.27 ± 0.19

^a^ Compounds **1** and **2A**–**2J** had no suppressive effects on BC cells at a concentration of 100 μM. ^b^ Doxorubicin (Dox) was used as positive control. * *p* < 0.05, *** *p* < 0.001 vs. PTE compound (Student’s *t* test).

## Data Availability

Not applicable.

## Supplementary Materials

The following supporting information can be downloaded at: https://www.mdpi.com/article/10.3390/ijms241411468/s1.
