# Supplementary material for: Synthesis, In Vitro, and In Vivo Investigations of Pterostilbene-Tethered Analogues as Anti-Breast Cancer Candidates
Source: Int J Mol Sci. 2023 Jul 14;24(14):11468. doi: 10.3390/ijms241411468 (PMC10380385; doi:10.3390/ijms241411468)
Supplement: Supplementary file 1 [file ijms-24-11468-s001.zip › ijms-2474349-supplementary.pdf]

*Supporting Information*

# Synthesis, In Vitro, and In Vivo Investigations of Pterostilbene-Tethered Analogues as Anti-Breast Cancer Candidates

**Guoxun Li [1], Jian Li [1,2], Wenqian Wang [1], Xiaoqing Feng [1], Xingkang Yu [1], Shuo Yuan [1], Wei Zhang [1], Jialing Chen [1] and Caijuan Hu [1,*]**

1 School of Pharmacy, Changzhou University, Changzhou 213164, China; liguoxuner@126.com (G.L.); lijianchem@cczu.edu.cn (J.L.); 15695186052@163.com (W.W.); fxqfw@cczu.edu.cn (X.F.); s21080917007@smail.cczu.edu.cn (X.Y.); s21090860003@smail.cczu.edu.cn (S.Y.); weiiiiii02212023@163.com (W.Z.); 19851990167@163.com (J.C.)

2 Jiangsu Key Laboratory of Advanced Catalytic Materials and Technology, Analysis and Testing Center, NERC Biomass of Changzhou University, Changzhou 213164, China

* Correspondence: hcj@cczu.edu.cn

## Supplementary Material Contents:

$^{1}$H NMR and $^{13}$C NMR spectra of target compounds

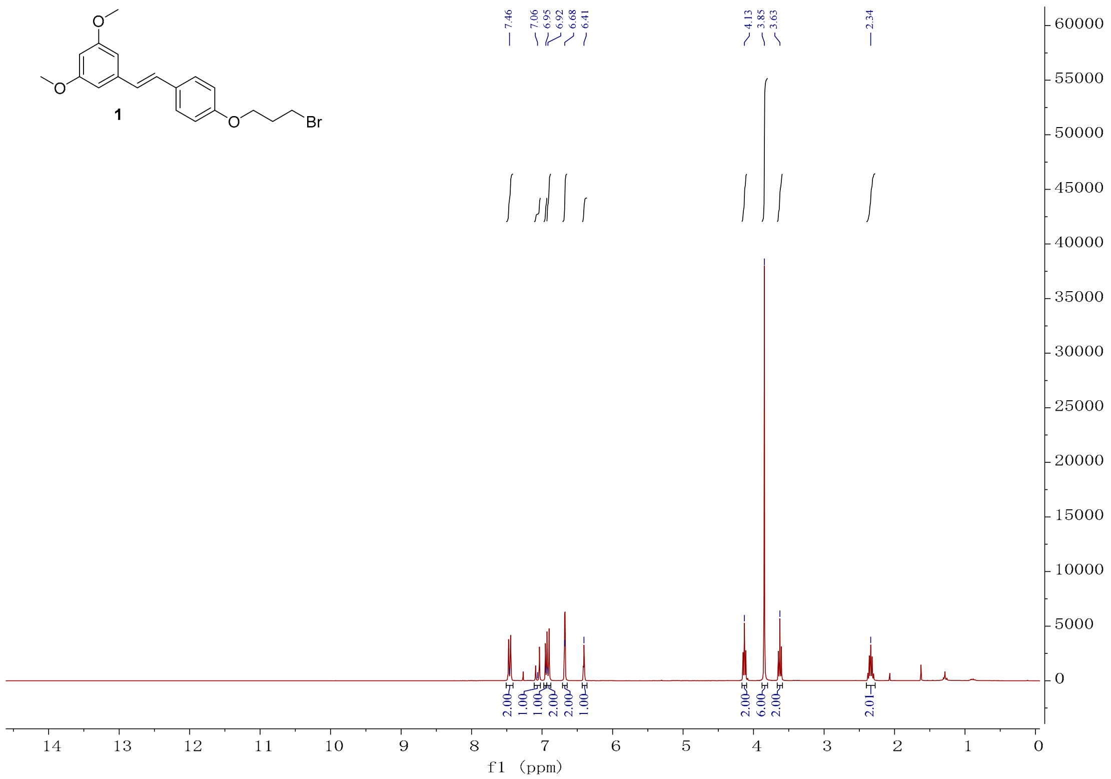


Figure S1. $^{1}$H NMR spectrum of compound **1**.

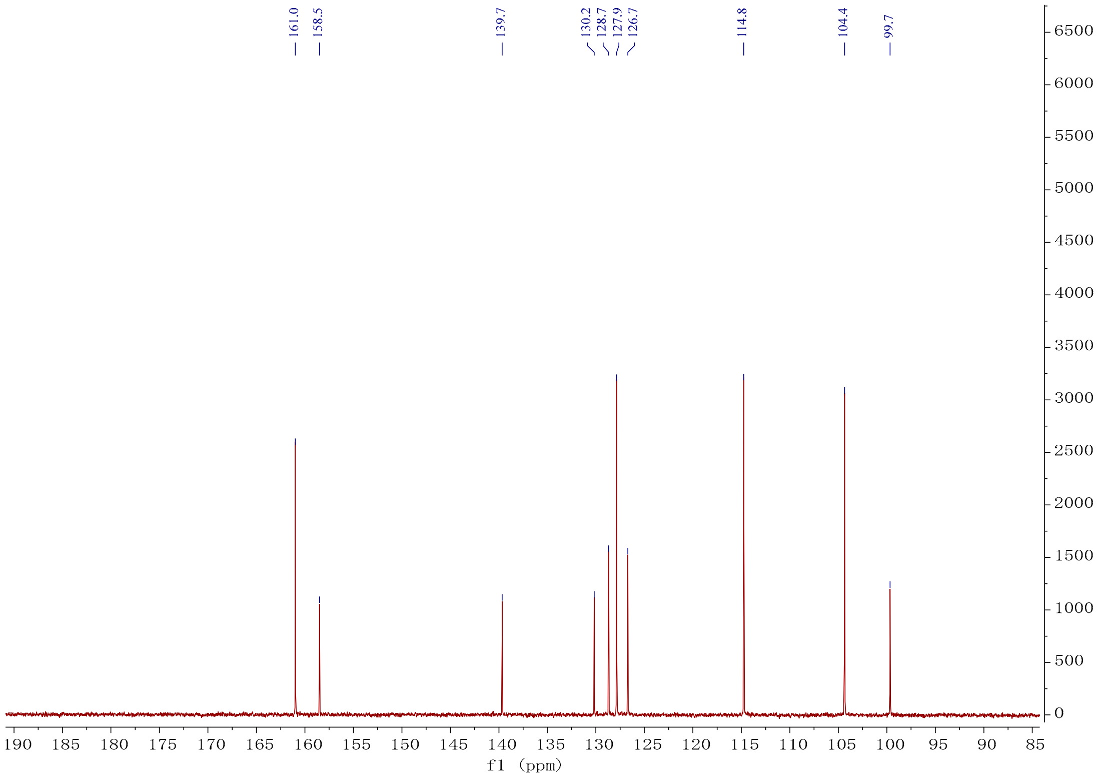


Figure S2. $^{13}$C NMR spectrum of compound **1**.

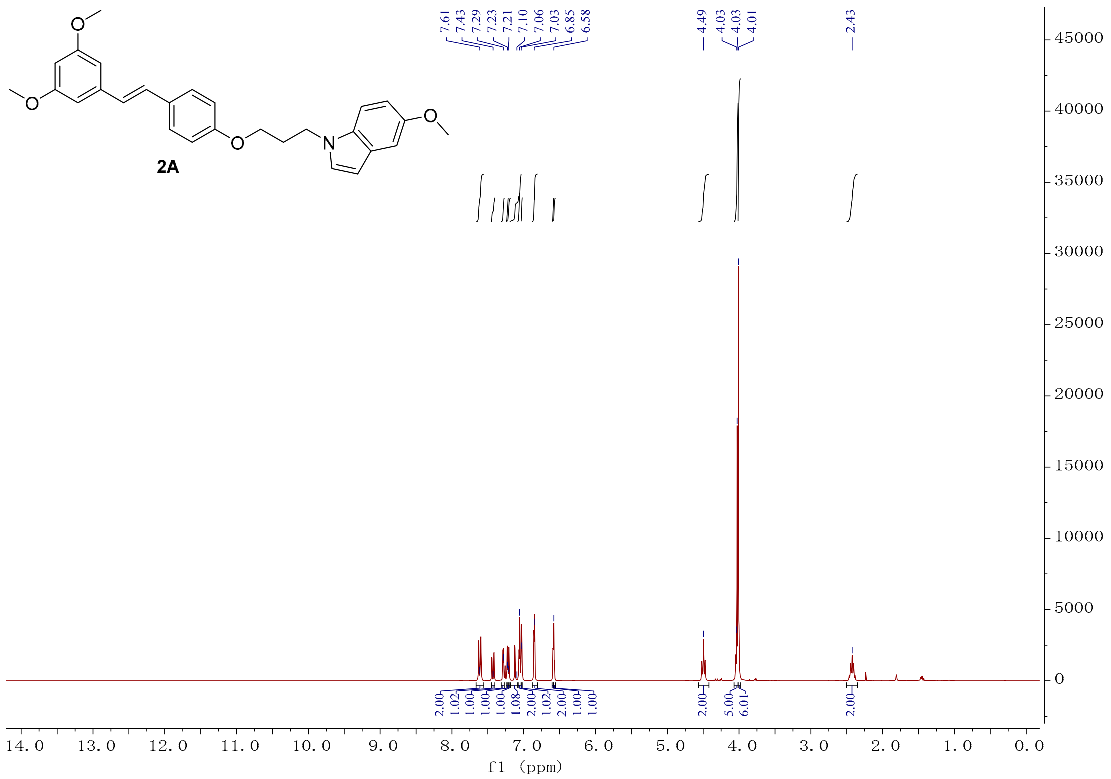


Figure S3. $^{1}$H NMR spectrum of compound **2A**.

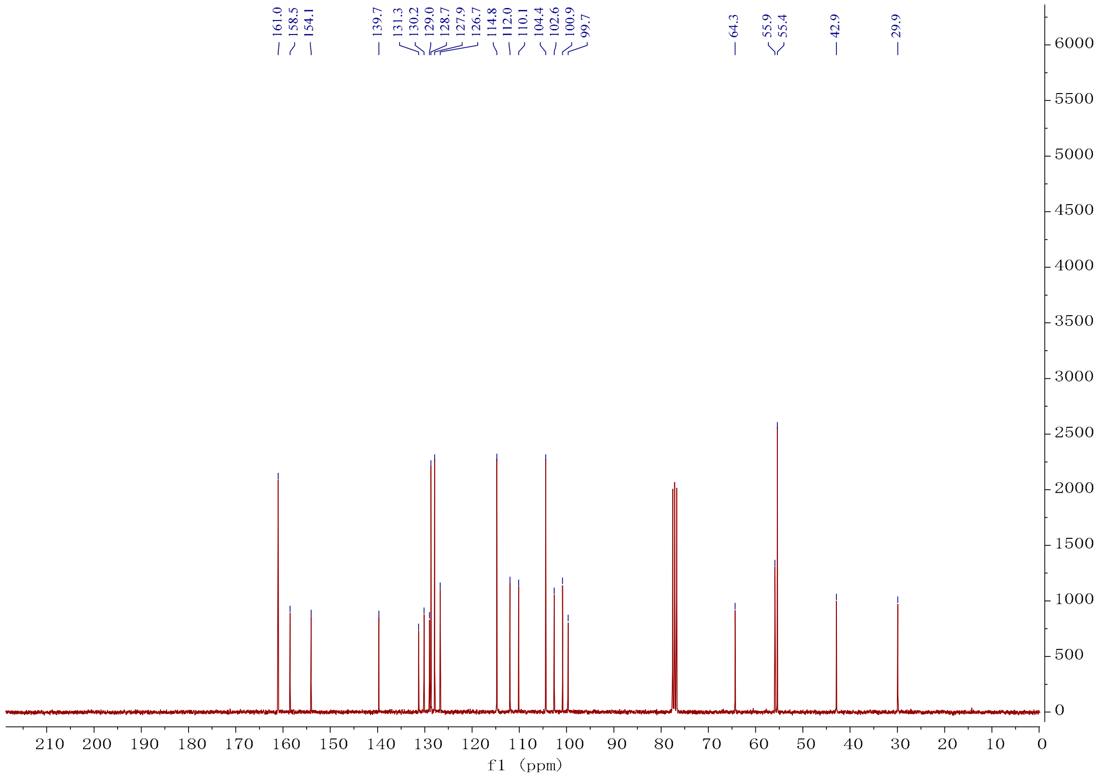


Figure S4. $^{13}$C NMR spectrum of compound **2A**.

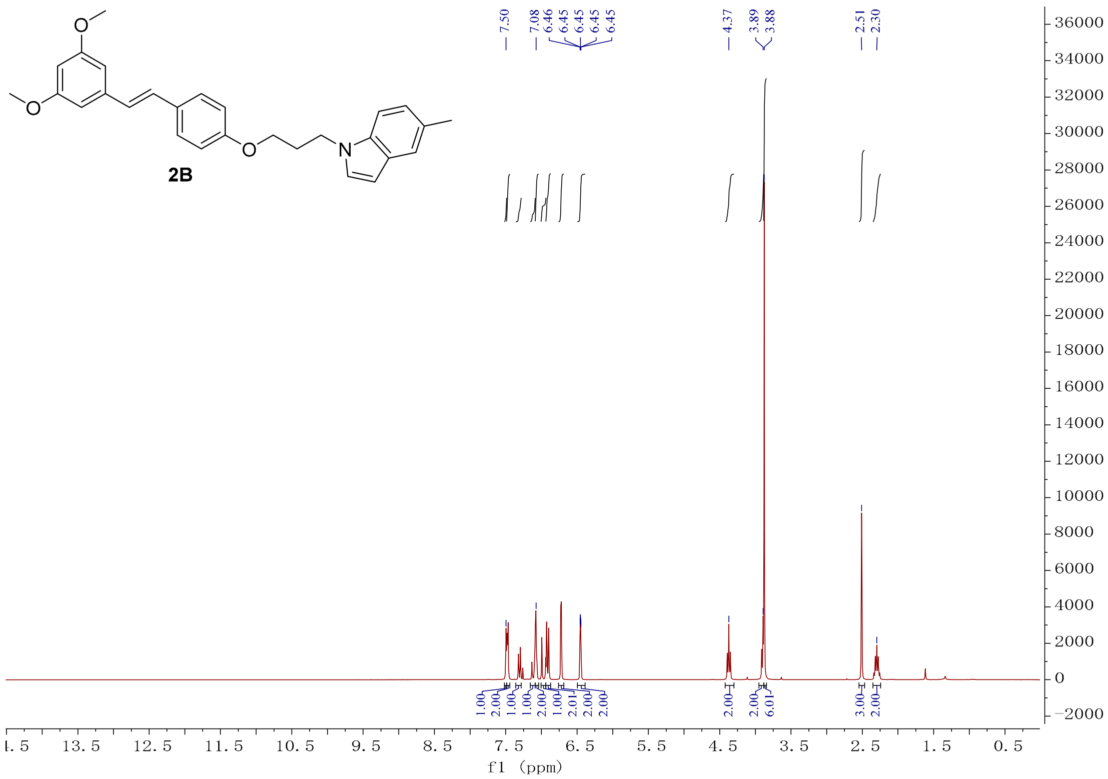


Figure S5. $^{1}$H NMR spectrum of compound **2B**.

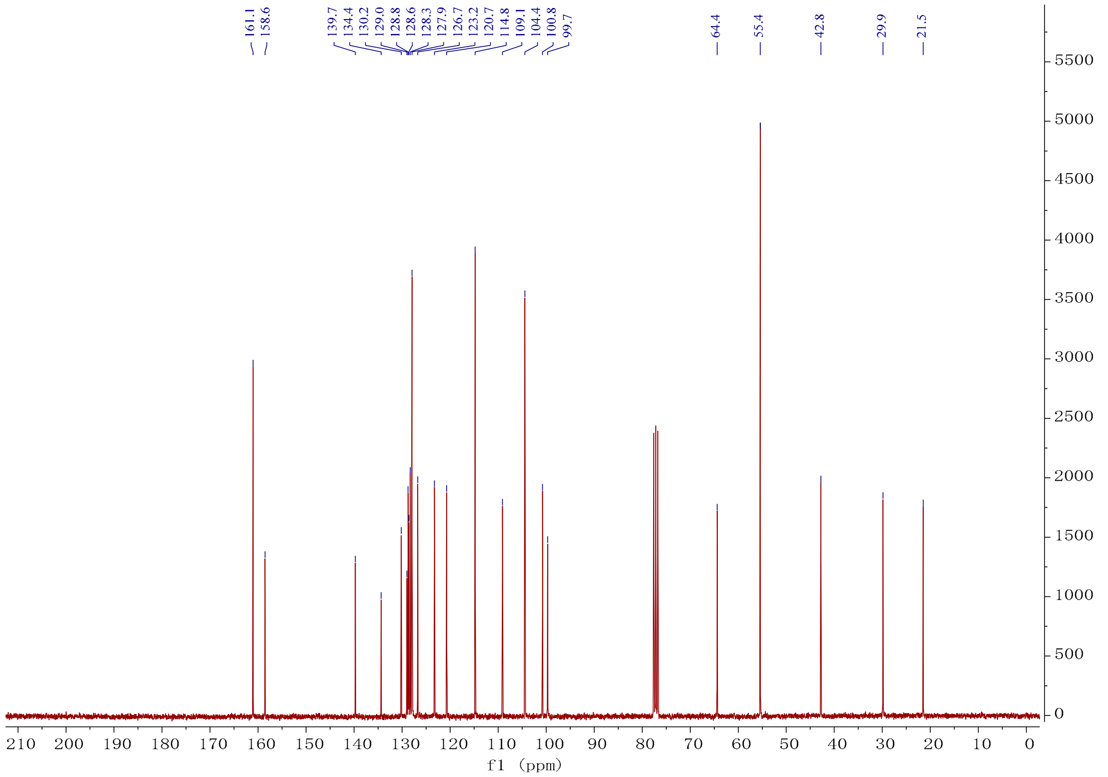


Figure S6. $^{13}$C NMR spectrum of compound **2B**.

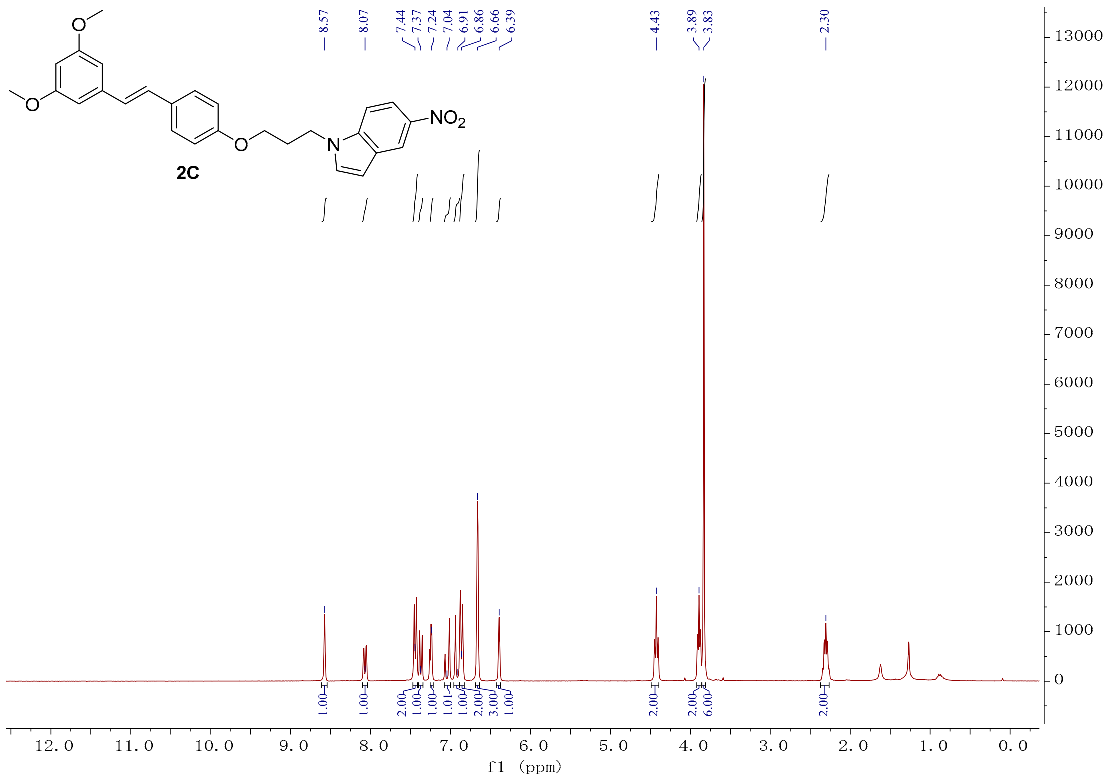

Figure S7. $^{1}$H NMR spectrum of compound **2C**.

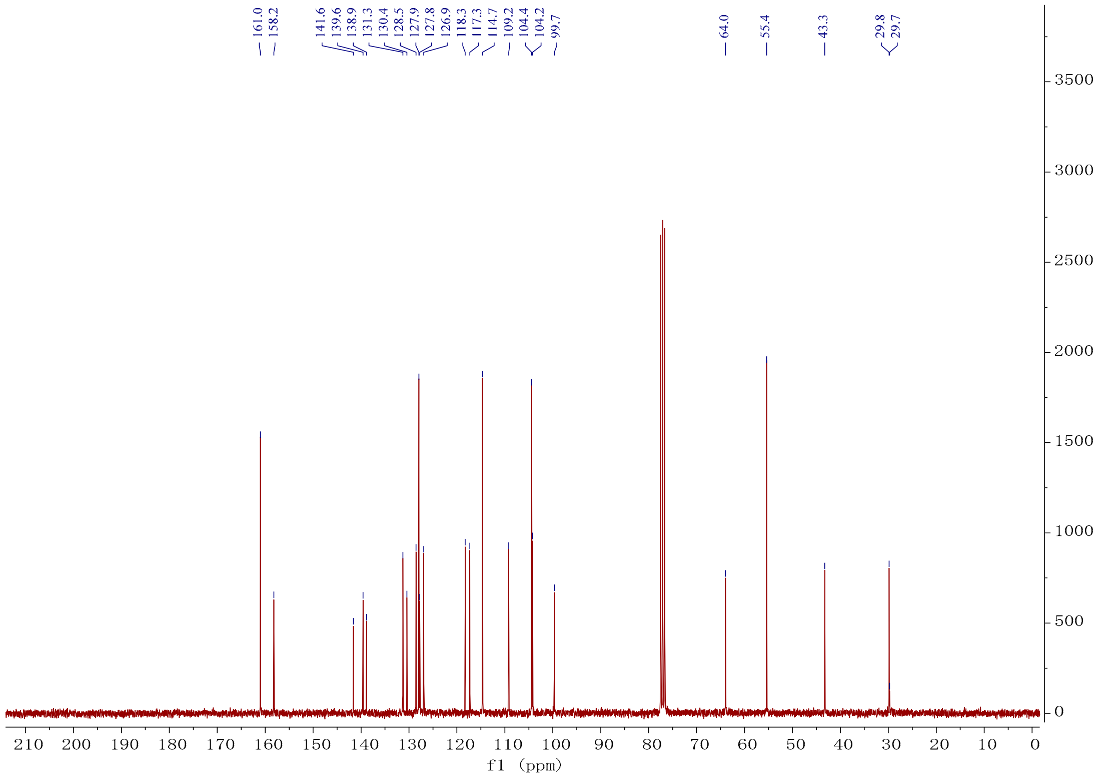

Figure S8. $^{13}$C NMR spectrum of compound **2C**.

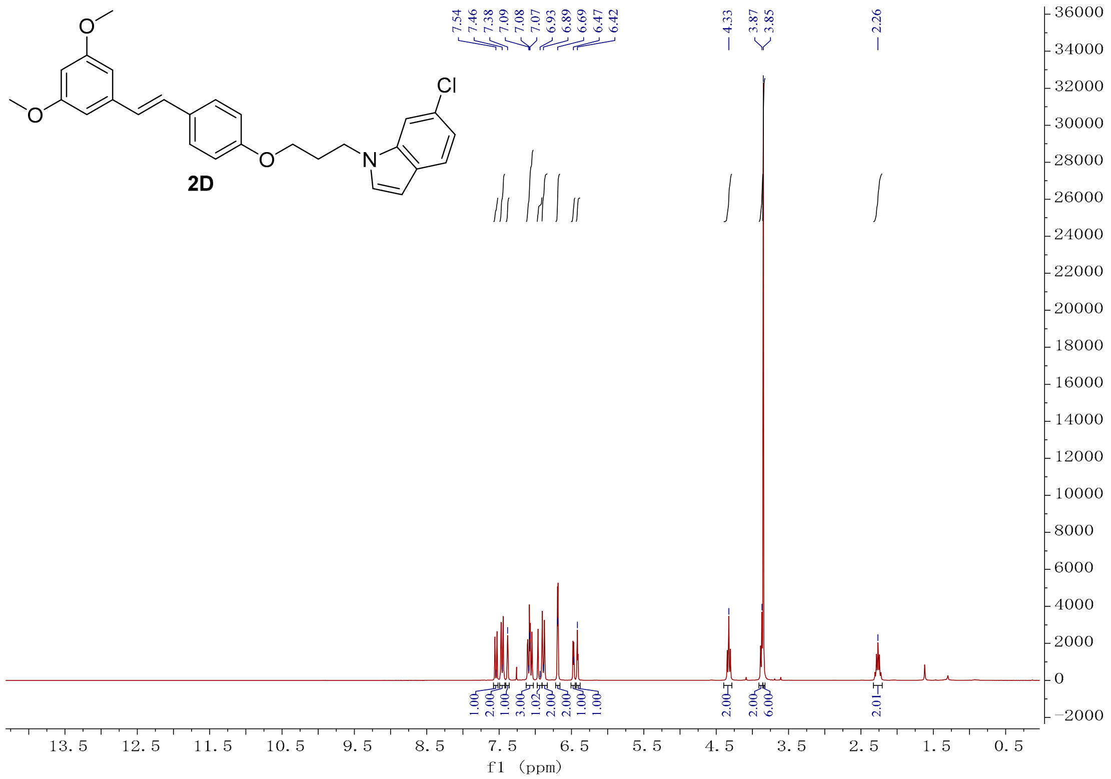


Figure S9. $^{1}$H NMR spectrum of compound **PTE-2D**.

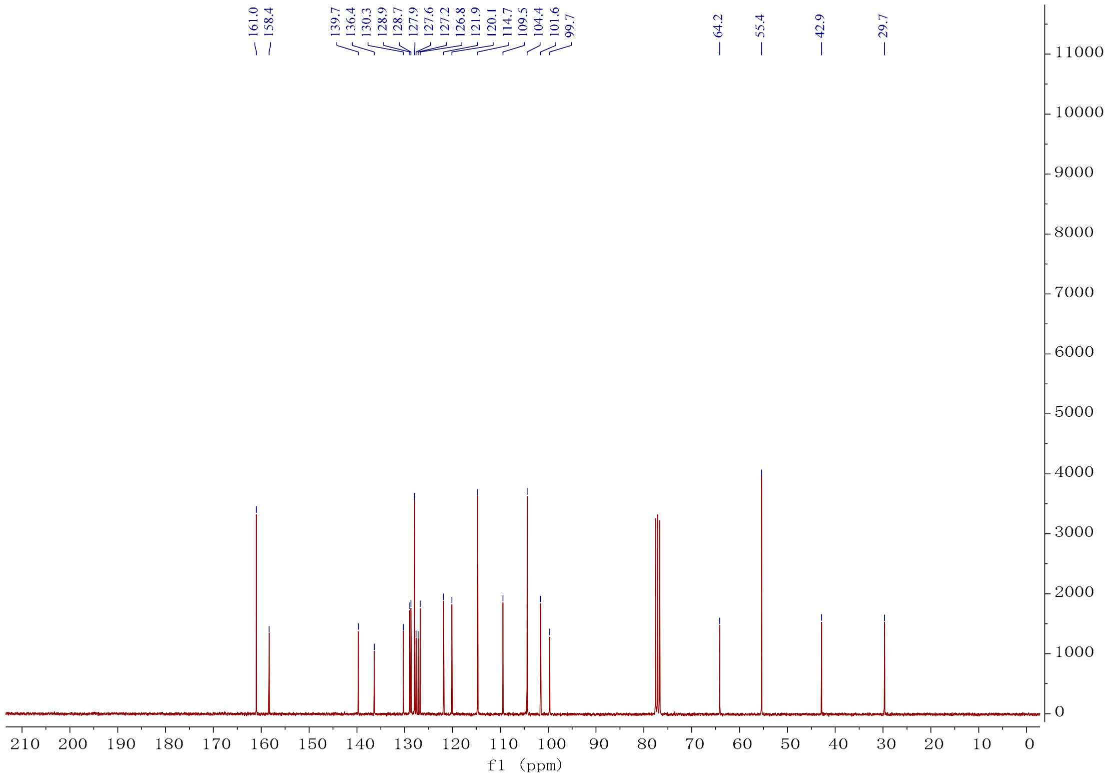


Figure S10. $^{13}$C NMR spectrum of compound **PTE-2D**.

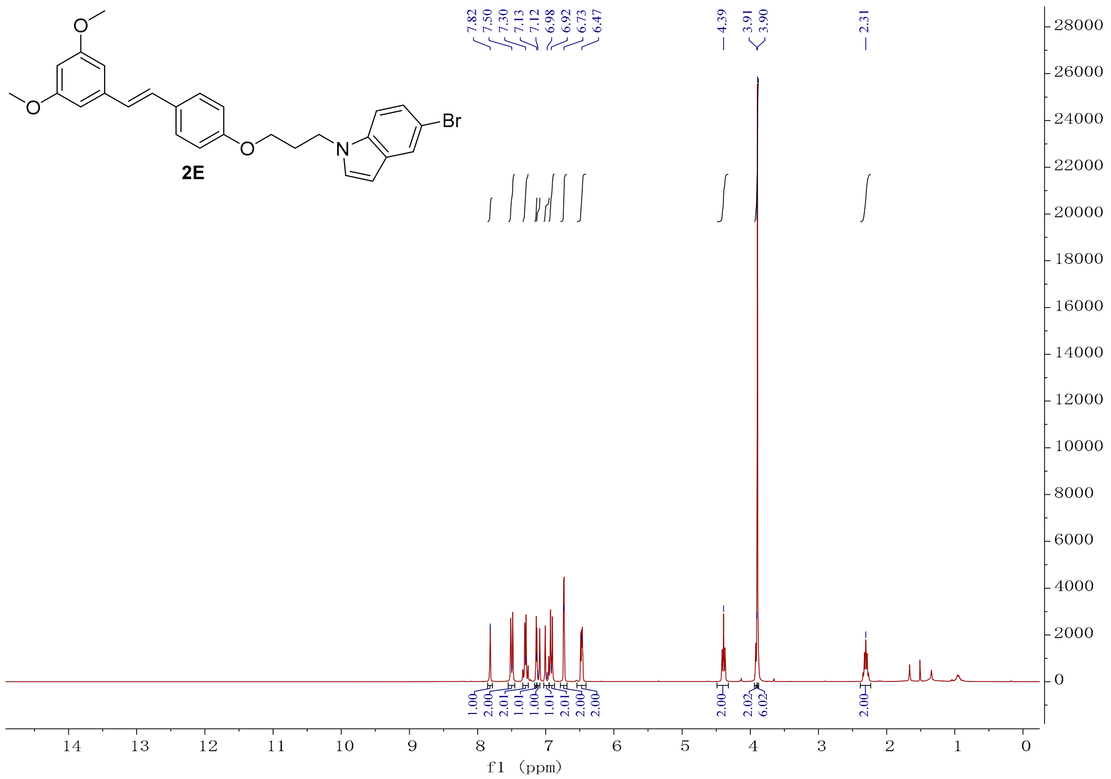


Figure S11. $^{1}$H NMR spectrum of compound **2E**.

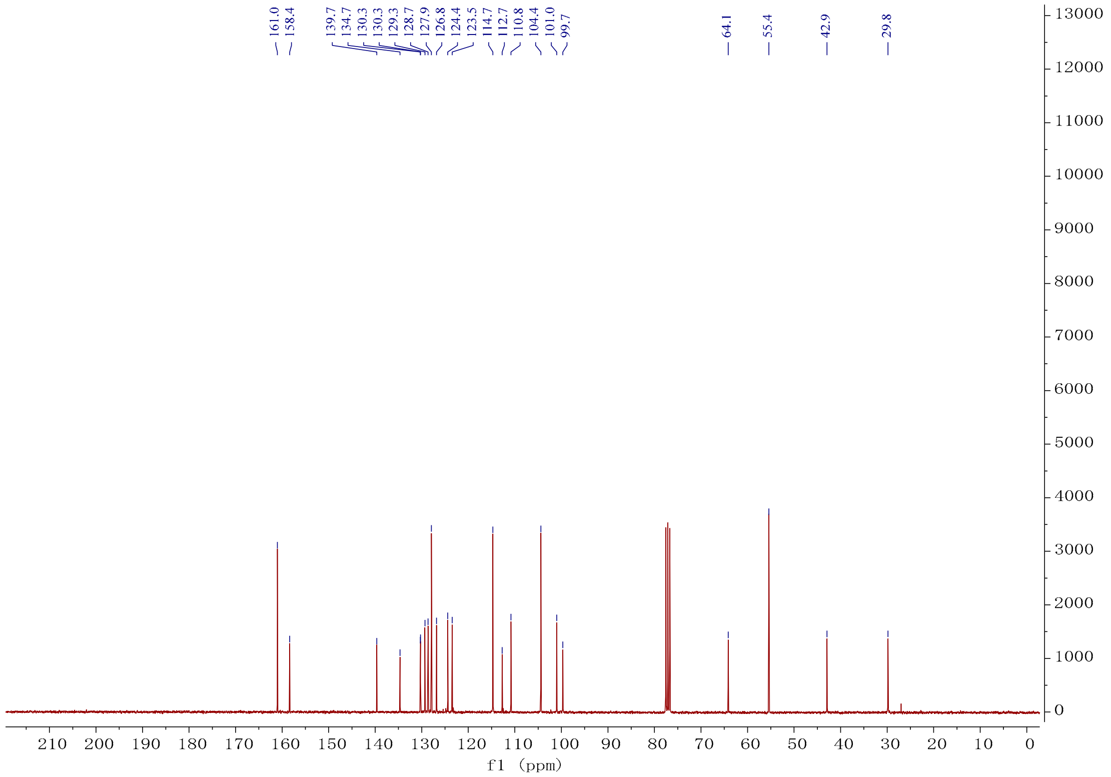


Figure S12. $^{13}$C NMR spectrum of compound **2E**.

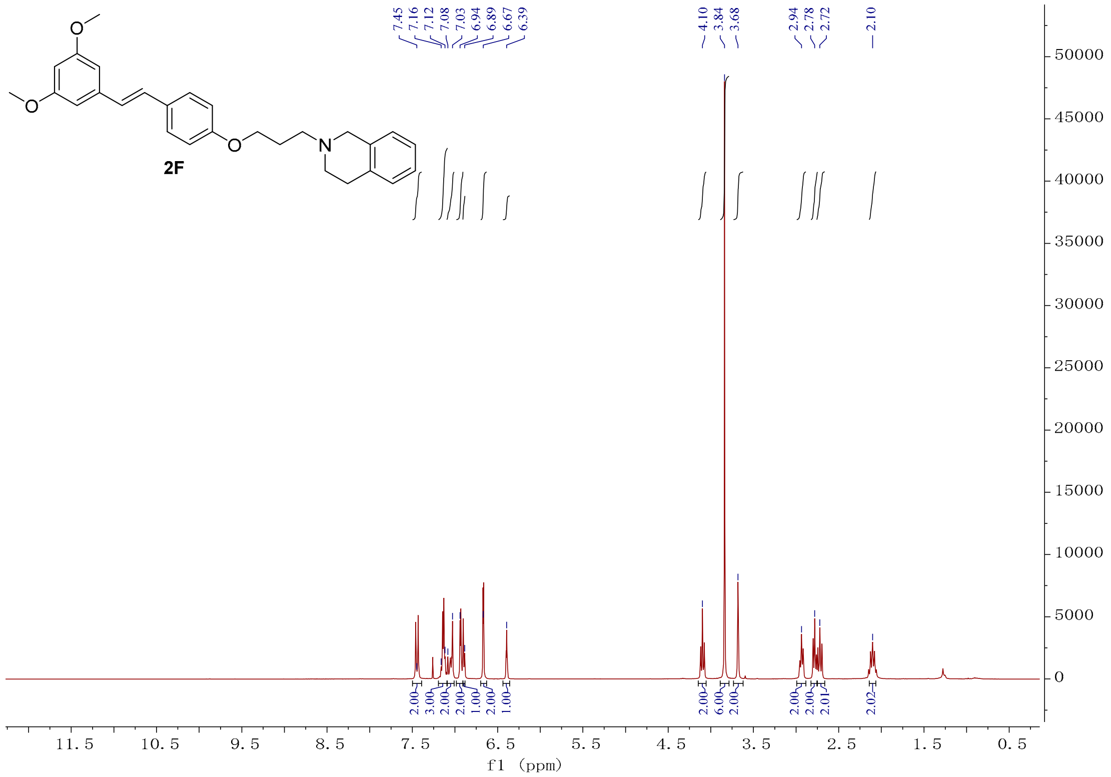


Figure S13. $^{1}$H NMR spectrum of compound **2F**.

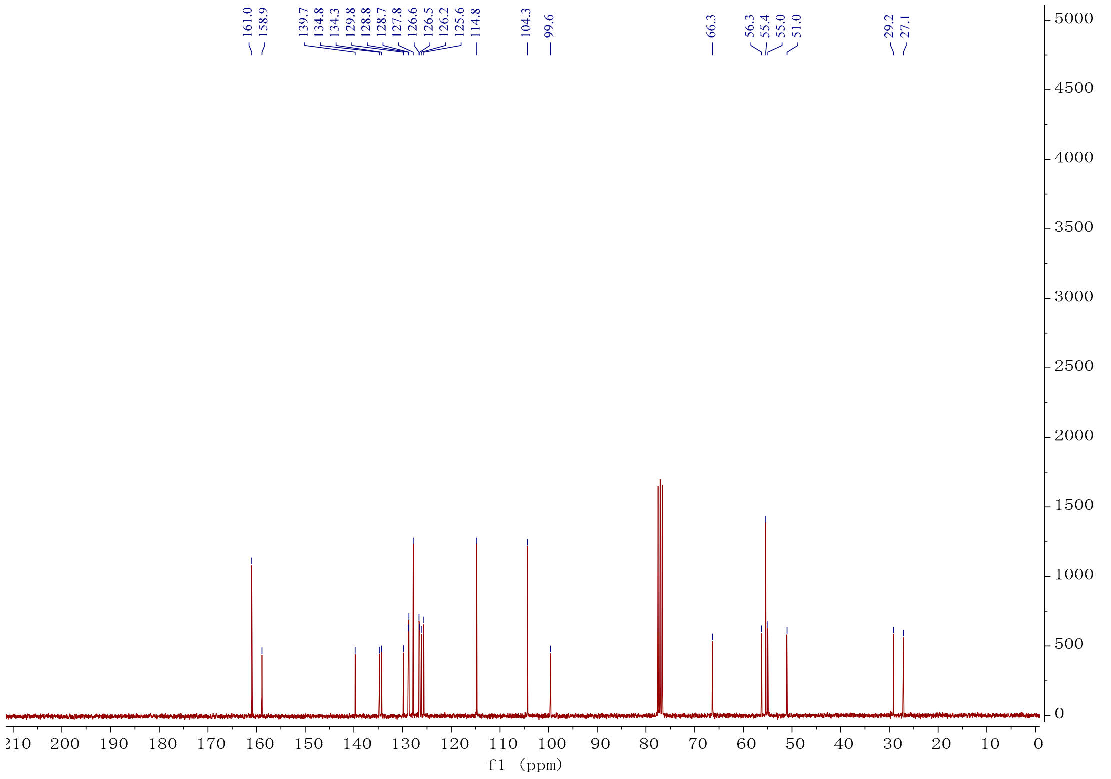


Figure S14. $^{13}$C NMR spectrum of compound **2F**.

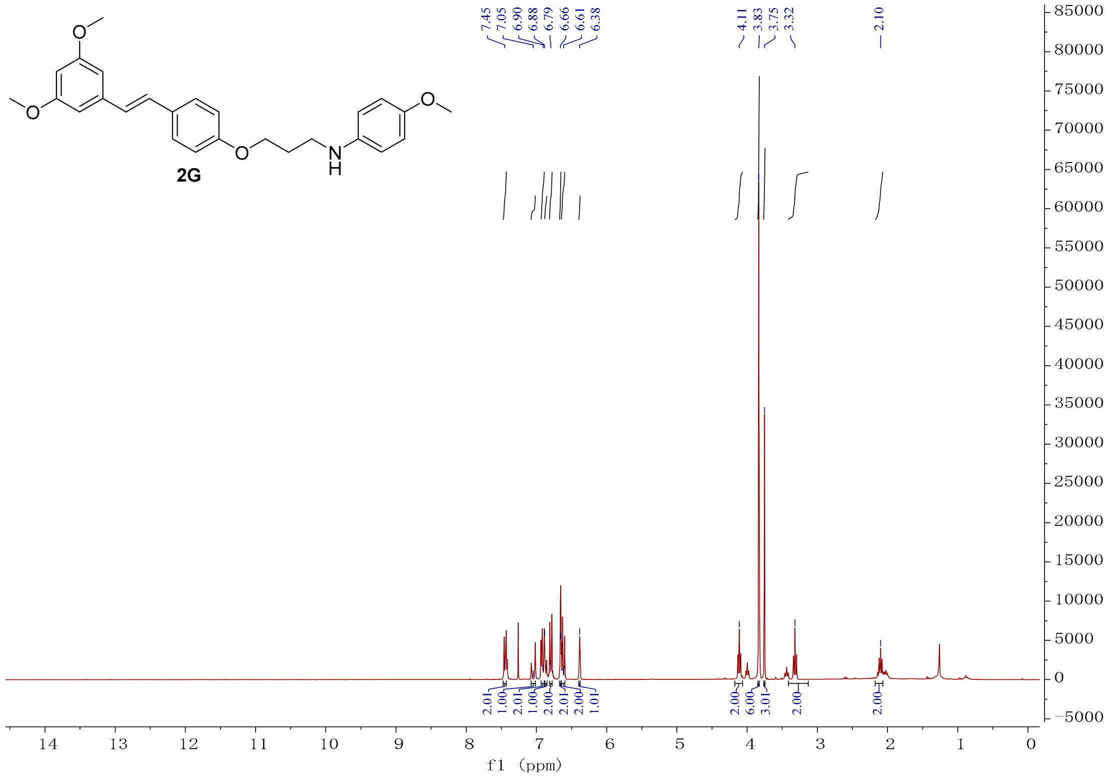


Figure S15. $^{1}$H NMR spectrum of compound **2G**.

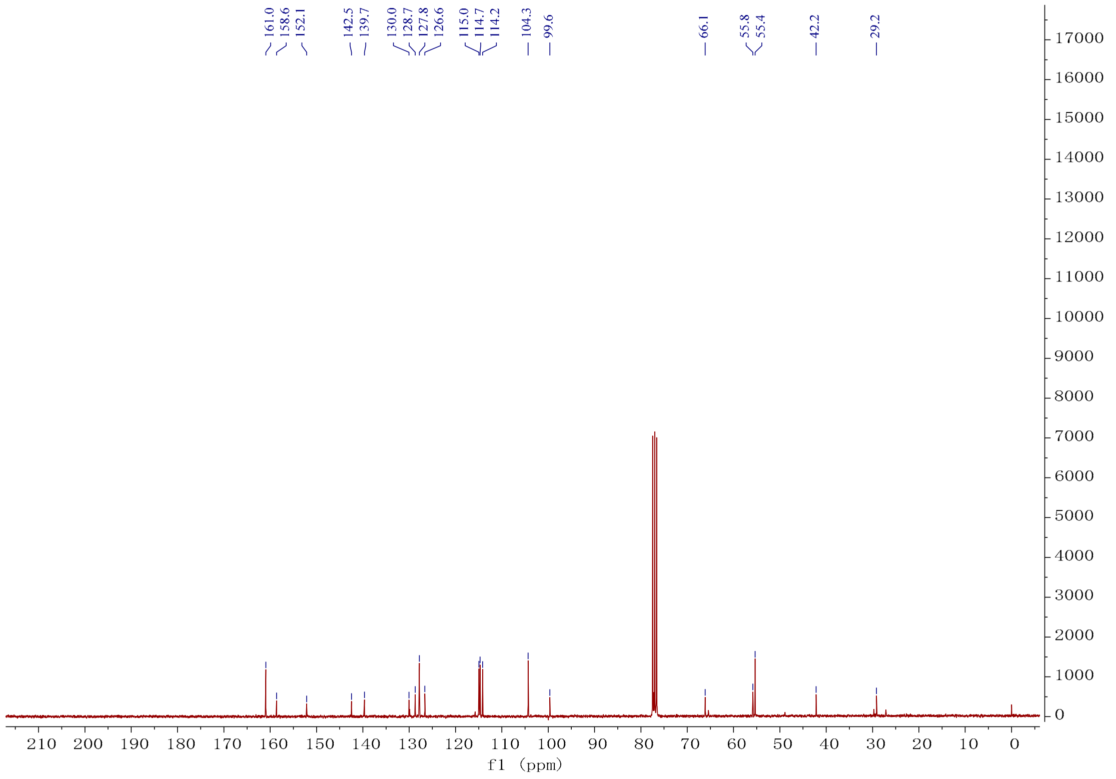


Figure S16. $^{13}$C NMR spectrum of compound **2G**.

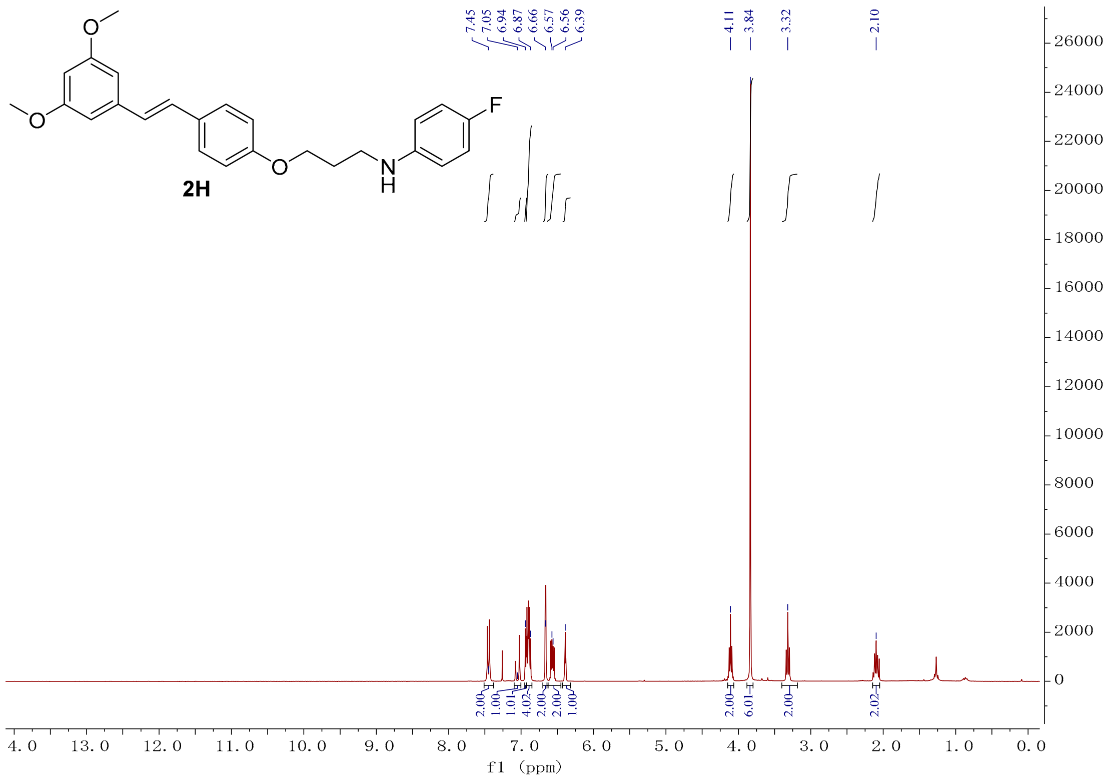


Figure S17. $^{1}$H NMR spectrum of compound **2H**.

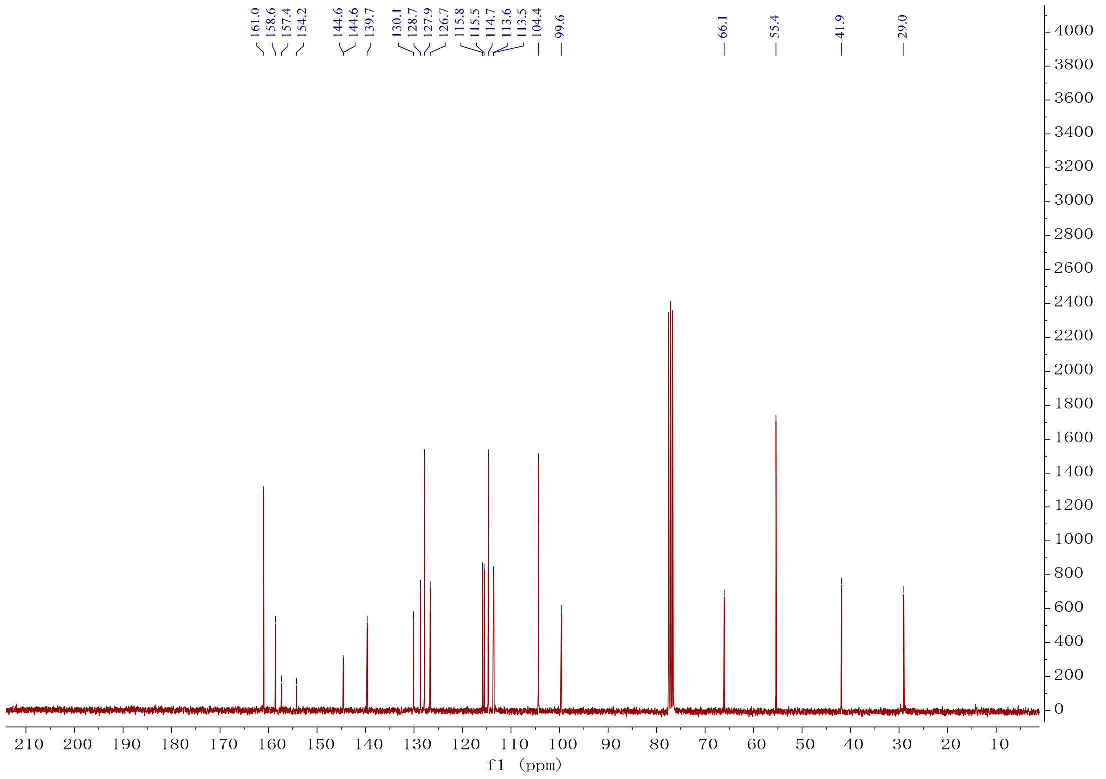


Figure S18. $^{13}$C NMR spectrum of compound **2H**.

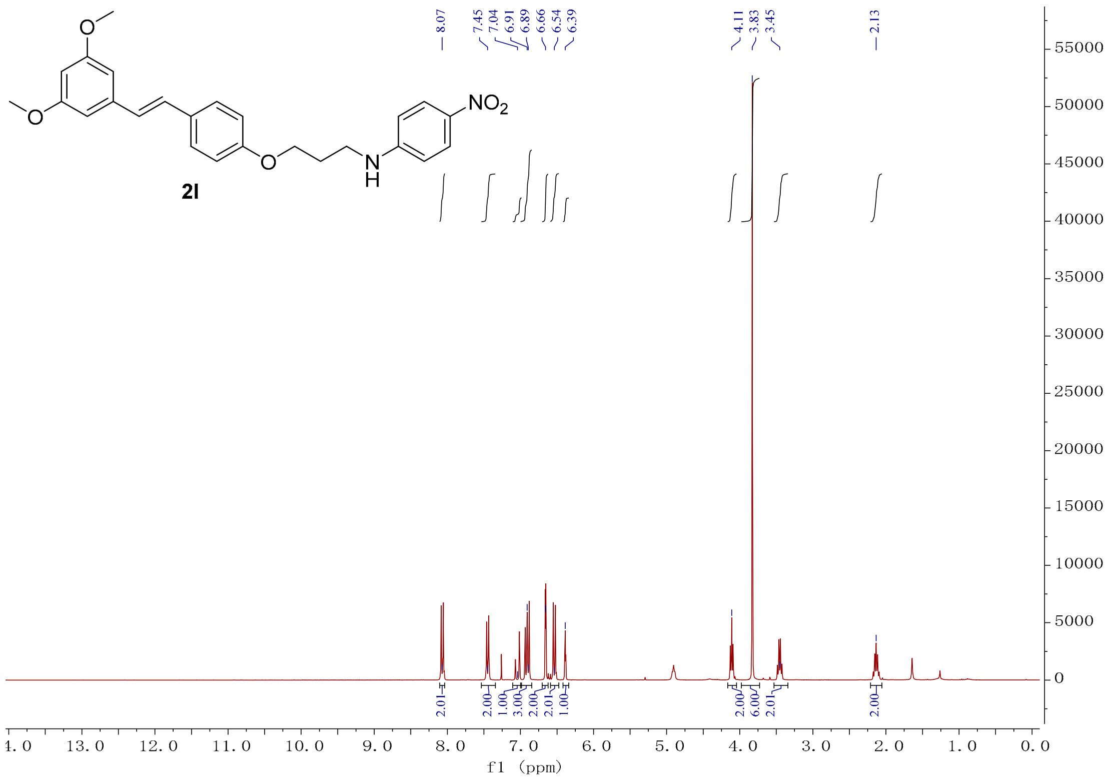


Figure S19. $^{1}$H NMR spectrum of compound **2I**.

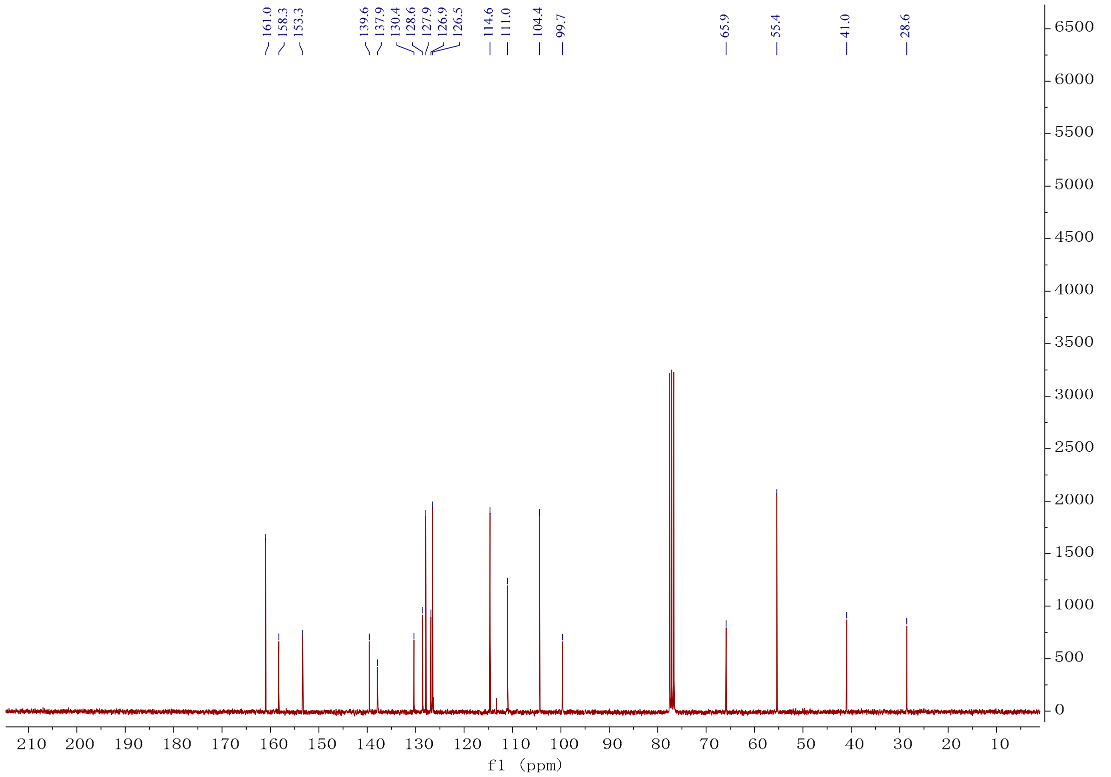


Figure S20. $^{13}$C NMR spectrum of compound **2I**.

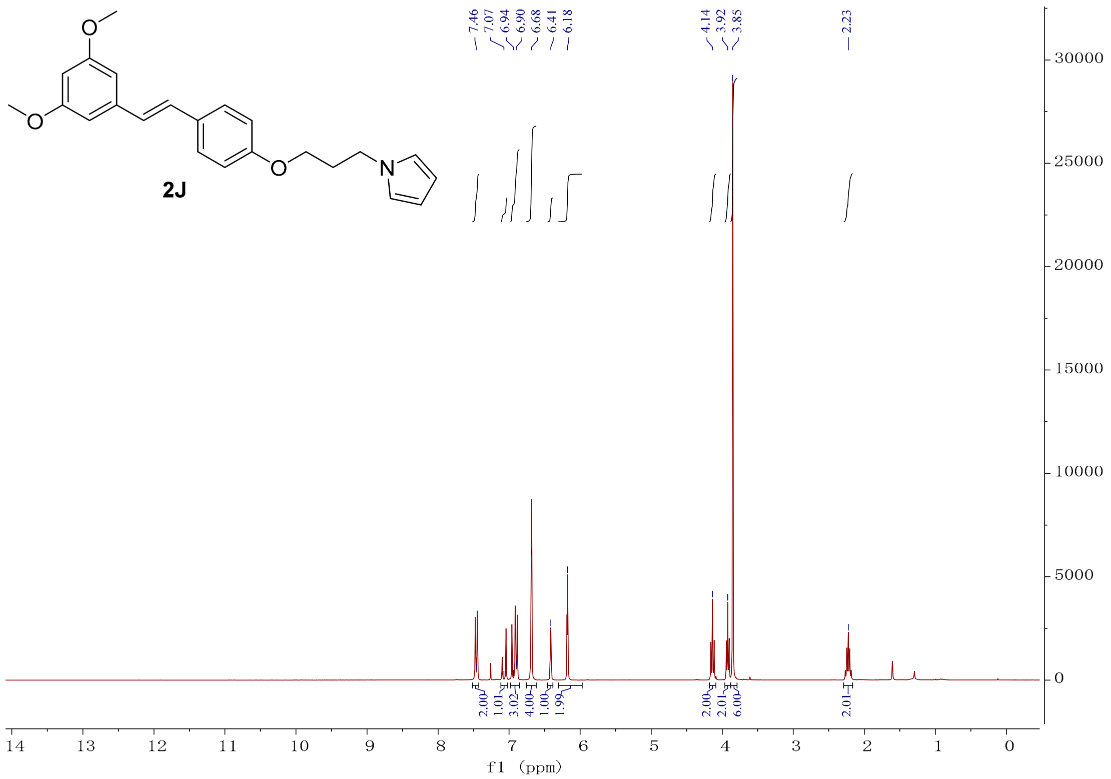


Figure S21. $^{1}$H NMR spectrum of compound **2J**.

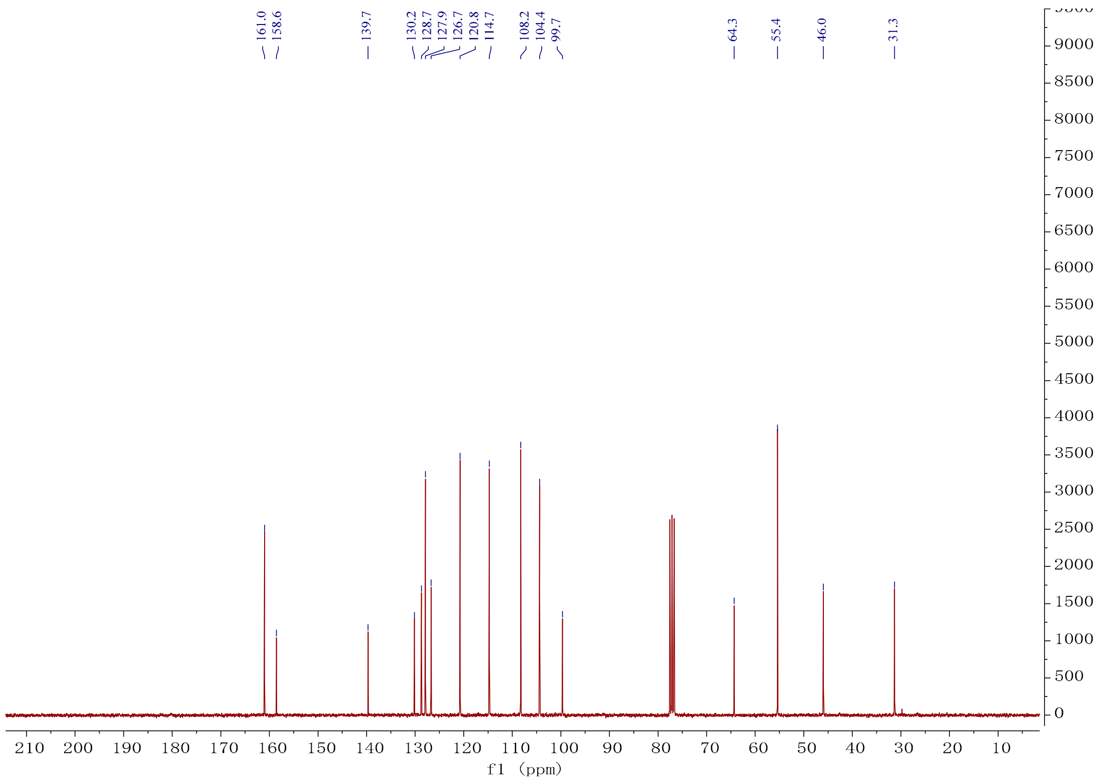


Figure S22. $^{13}$C NMR spectrum of compound **2J**.

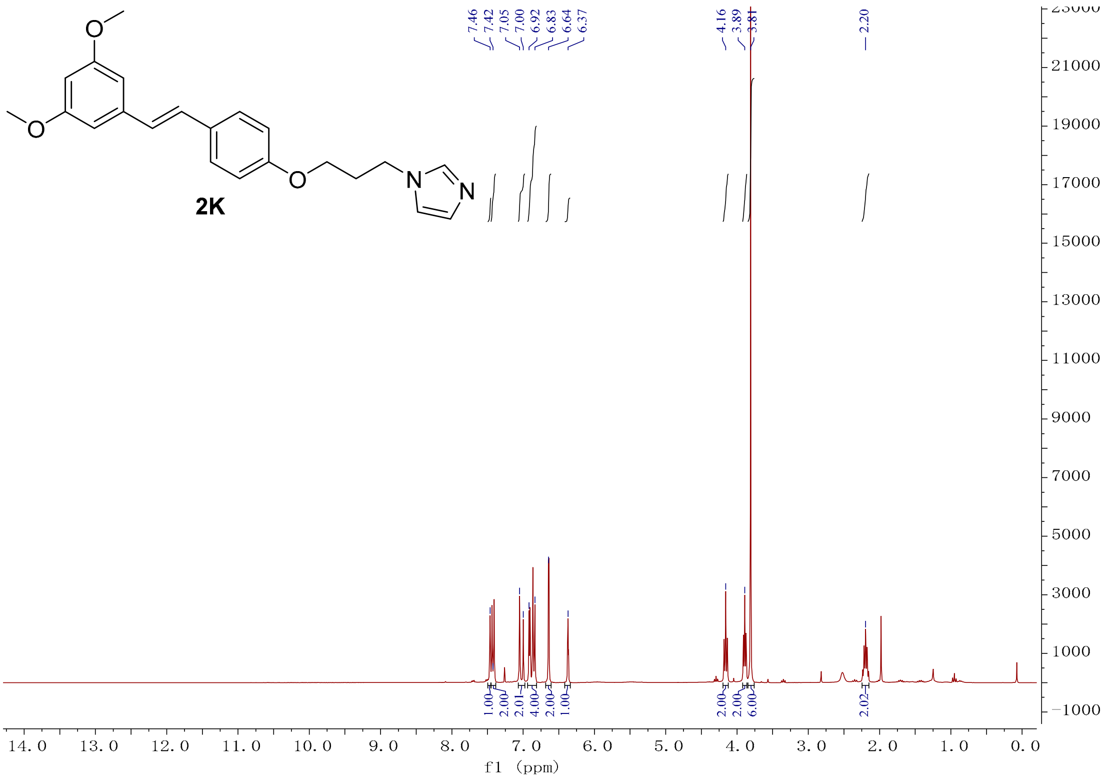


Figure S23. $^{1}$H NMR spectrum of compound **2K**.

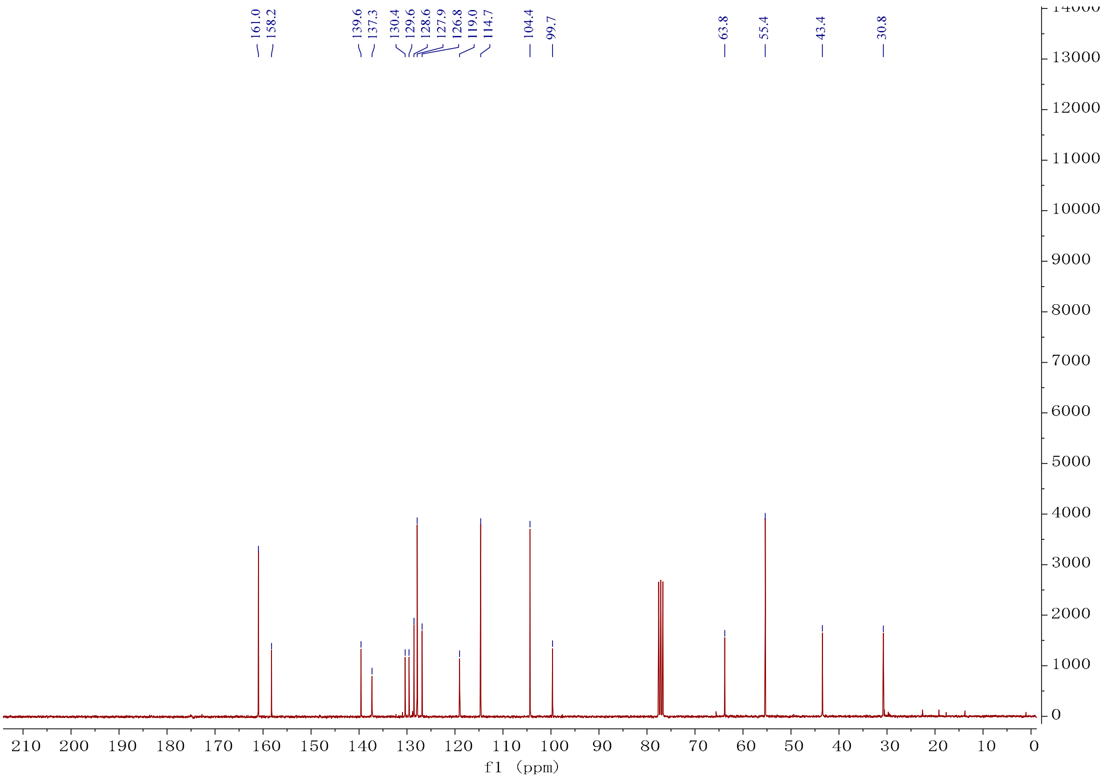


Figure S24. $^{13}$C NMR spectrum of compound **2K**.

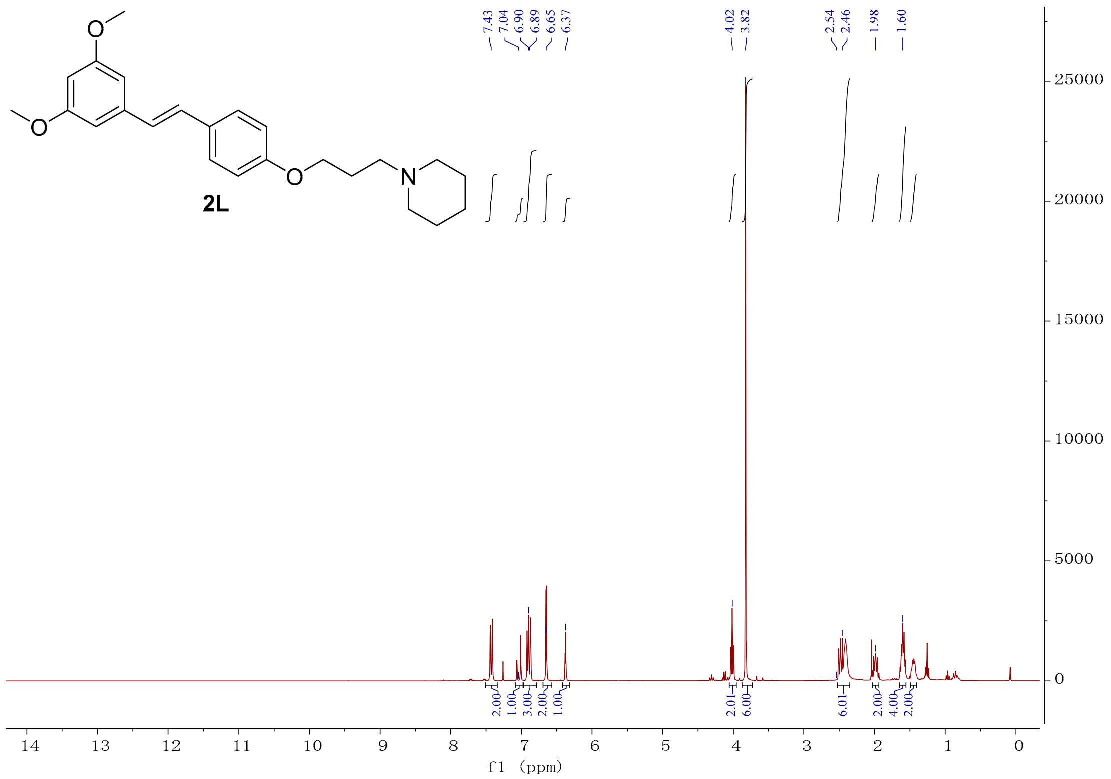


Figure S25. $^{1}H$ NMR spectrum of compound **2L**.

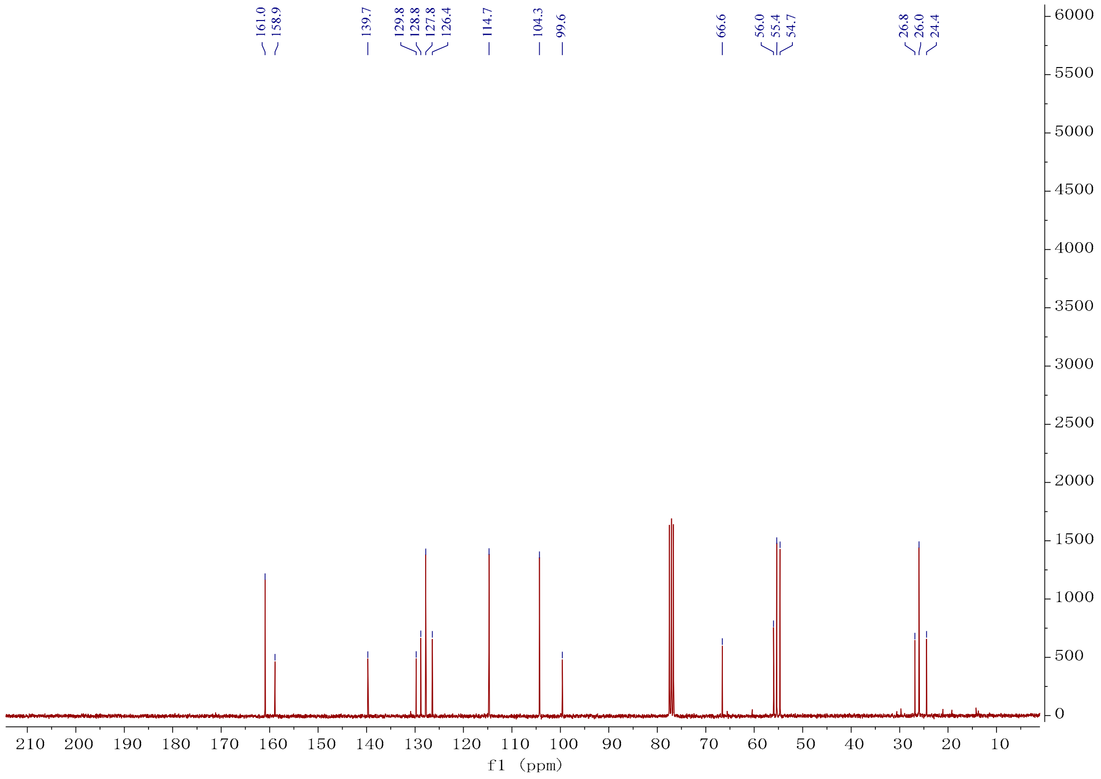


Figure S26. $^{13}C$ NMR spectrum of compound **2L**.

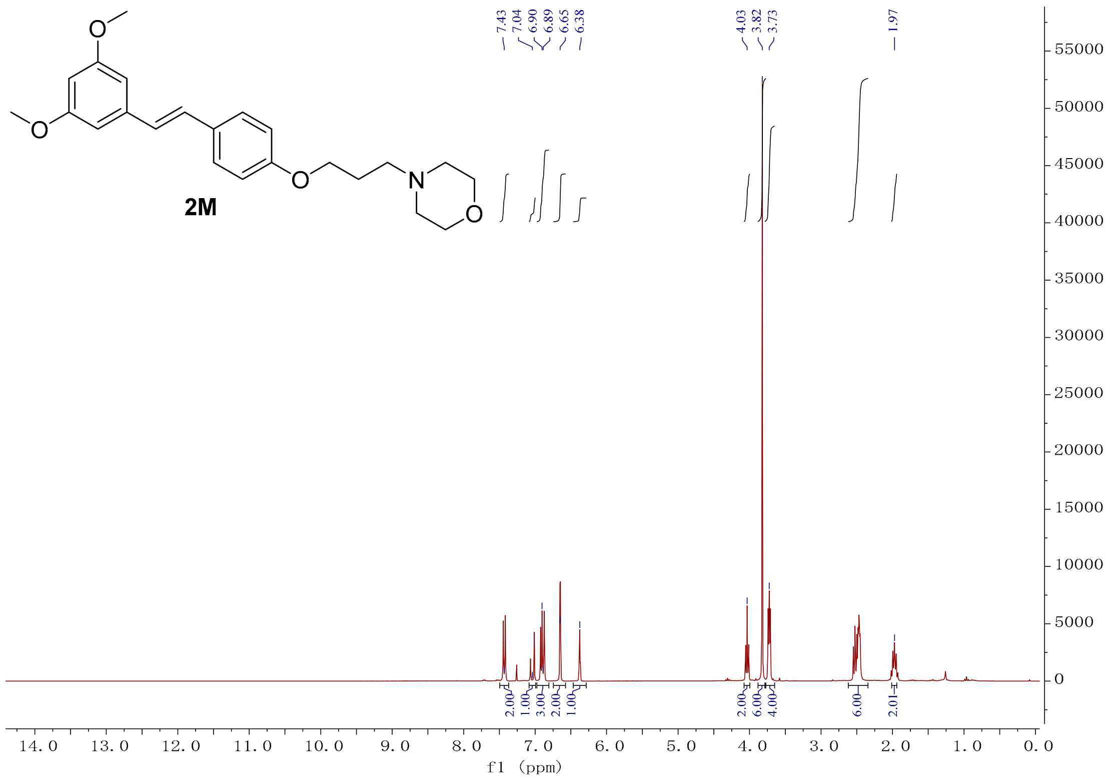

Figure S27. $^{1}$H NMR spectrum of compound **2M**.

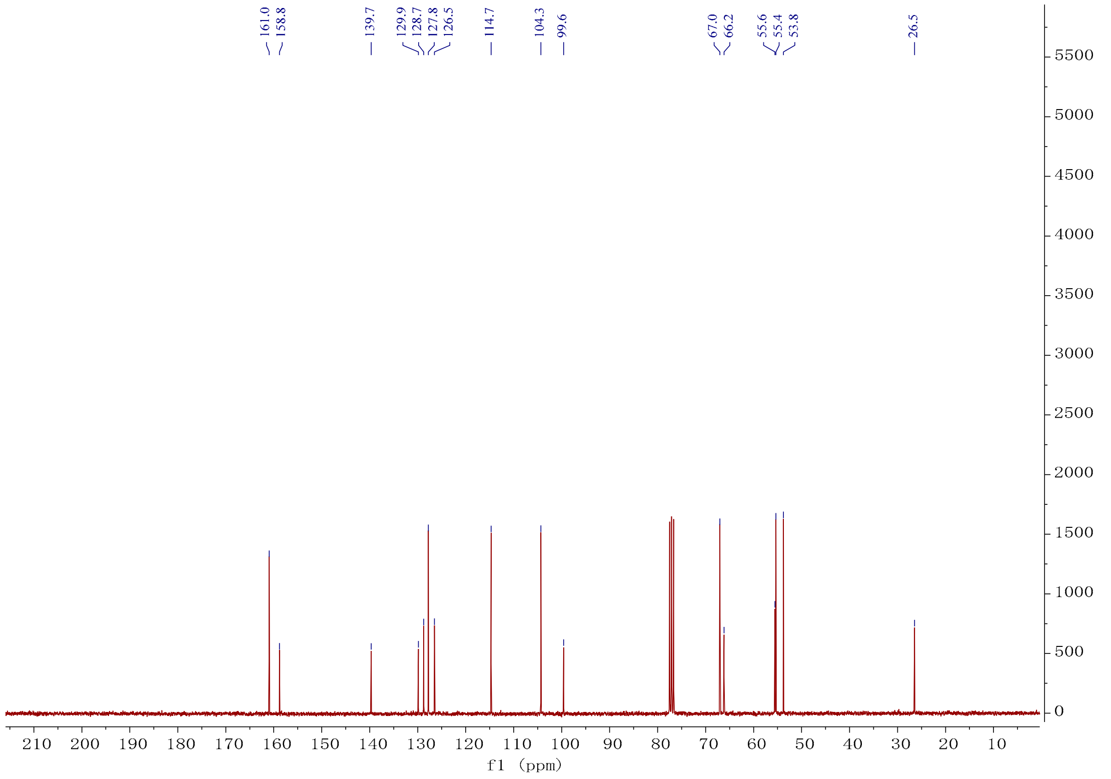

Figure S28. $^{13}$C NMR spectrum of compound **2M**.

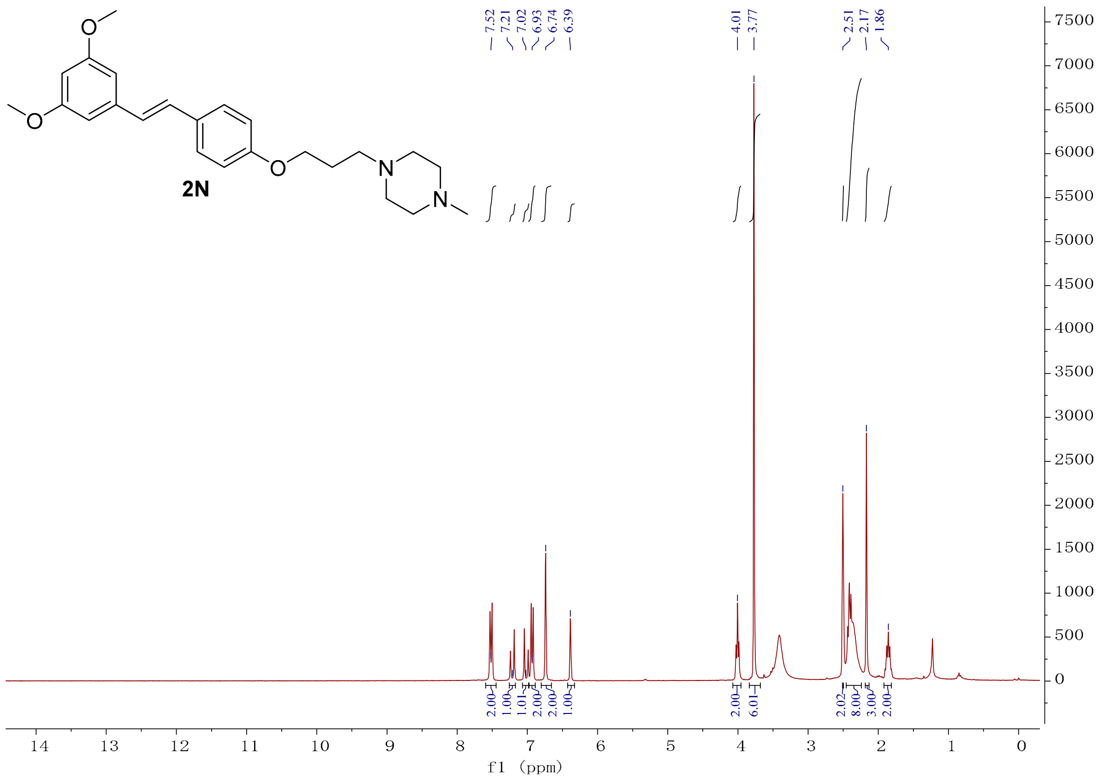


Figure S29. $^{1}$H NMR spectrum of compound **2N**.

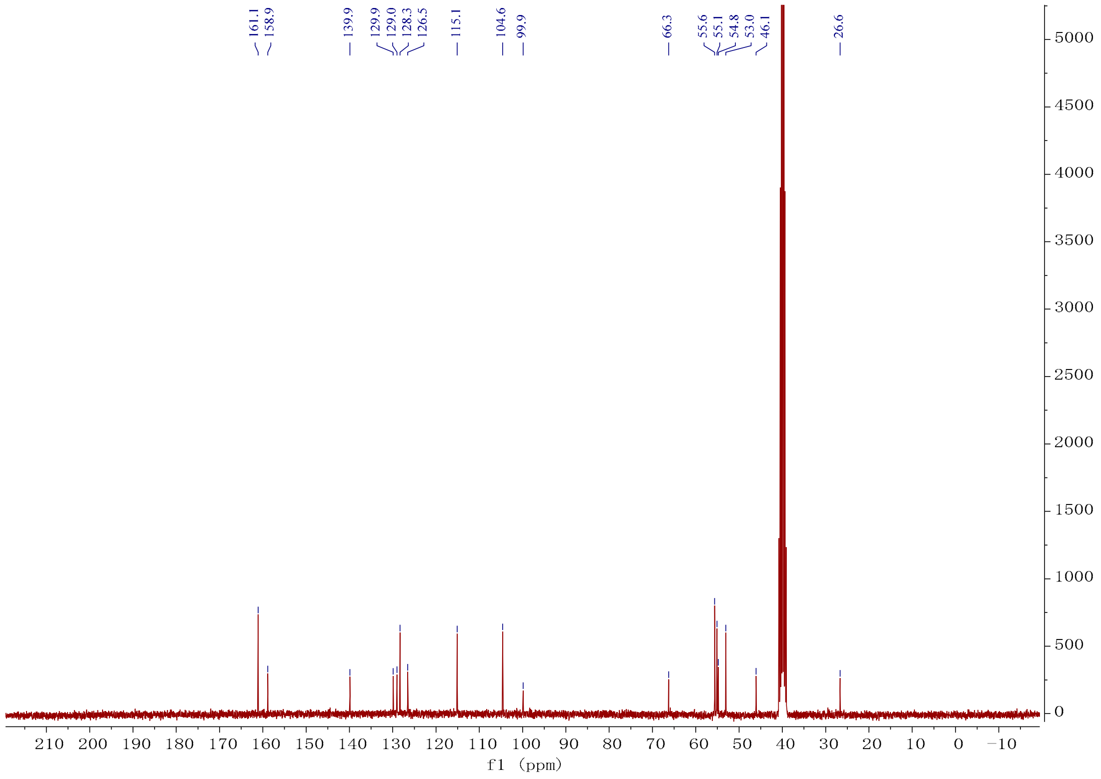


Figure S30. $^{13}$C NMR spectrum of compound **2N**.
